# ARID1A deficiency promotes progression and potentiates therapeutic antitumour immunity in hepatitis B virus-related hepatocellular carcinoma

**DOI:** 10.1186/s12876-023-03059-w

**Published:** 2024-01-02

**Authors:** Tao Xing, Li Li, Xiaosong Rao, Jing Zhao, Yiran Chen, Gaoda Ju, Yaping Xu, Xuan Gao, Guilan Dong, Xuefeng Xia, Yanfang Guan, Lingling Zhang, Zhenping Wen, Jun Liang

**Affiliations:** 1 Departments of Oncology, Peking University International Hospital, 1 Life Park Road, Life Science Park of Zhongguancun, Changping, Beijing, 102206 China; 2 Key Laboratory of Carcinogenesis and Translational Research (Ministry of Education), Peking University Cancer Hospital & Institute, No. 52, Fucheng Road, Haidian District, Beijing, 100142 China; 3 HAINAN YILING Medical Industry Development Co.,Ldt, Qionghai, Hainan 571442 China; 4 Department of Pathology and Neuropathology, University Hospital Tübingen, Tübingen, 72074 Germany; 5 Department of Radiation Oncology, Fujian Medical University Cancer Hospital, Fujian Cancer Hospital, Fuzhou, 350014 China; 6 Geneplus-Beijing Institute, Beijing, 102206 China; 7 Tangshan People’s Hospital, Tangshan, Hebei 063001 China; 8 Inner Mongolia Cancer Hospital, 42 Zhaowuda Road, Saihan District, Hohhot, Inner Mongolia 010020 P. R. China

**Keywords:** ARID1A, Hepatocellular carcinoma, Immunotherapy, TMB, TIM3

## Abstract

**Background:**

Exploring predictive biomarkers and therapeutic strategies of ICBs has become an urgent need in clinical practice. Increasing evidence has shown that ARID1A deficiency might play a critical role in sculpting tumor environments in various tumors and might be used as pan-cancer biomarkers for immunotherapy outcomes. The current study aims to explored the immune-modulating role of ARID1A deficiency in Hepatitis B virus (HBV) related hepatocellular carcinoma (HBV-HCC) and its potential immunotherapeutic implications.

**Methods:**

In the current study, we performed a comprehensive analysis using bioinformatics approaches and pre-clinical experiments to evaluate the ARID1A regulatory role on the biological behavior, and immune landscape of Hepatitis B virus (HBV) related hepatocellular carcinoma (HBV-HCC). A total of 425 HBV-related hepatocellular carcinoma patients from TCGA-LIHC, AMC and CHCC-HBV cohort were enrolled in bioinformatics analysis. Immunohistochemical staining of HBV-HCC specimens and ARID1A deficiency cellular models were used to validate the results of the analysis.

**Results:**

Our results have shown that ARID1A deficiency promoted tumor proliferation and metastasis. More importantly, ARID1A deficiency in HBV-HCC was associated with the higher TMB, elevated immune activity, and up-regulated expression of immune checkpoint proteins, especially TIM-3 in HBV-HCC. Further, the expression of Galectin-9, which is the ligand of TIM-3, was elevated in the ARID1A knockout HBV positive cell line.

**Conclusion:**

To conclude, we have shown that the ARID1A deficiency was correlated with more active immune signatures and higher expression of immune checkpoints in HBV-HCC. Additionally, the present study provides insights to explore the possibility of the predictive role of ARID1A in HBV-HCC patients responsive to immunotherapy.

**Supplementary Information:**

The online version contains supplementary material available at 10.1186/s12876-023-03059-w.

## Introduction

Liver cancer is the sixth most prevalent cancer and the fourth leading cause of cancer-related deaths worldwide [1]. Hepatocellular carcinoma accounts for nearly 85–90% of liver cancer [2]. Immune checkpoint blockades (ICBs) have shown promising clinical success in many malignancies, including HCC [3, 4]. However, it benefits only a limited subset of patients, with less than a 20% response rate for PD-1 inhibitor monotherapy in HCC [5]. It was demonstrated that genomic alterations might dictate the phenotypic performance of tumours and influence the therapeutic sensitivity of the ICBs [6]. Hence, understanding the regulatory role of genomic alterations may provide novel insight for developing innovative biomarkers as well as therapeutic strategies for cancer. Meanwhile, next-generation sequencing (NGS) revealed that different genomic alterations between HCC with contrasting pathological features should be treated differently [7, 8]. Chronic infections with HBV are significant causes of HCC in sub-Saharan Africa and East Asia, which is entirely different from western countries [9]. Therefore, we have mainly focused on HBV-HCC in the current study.

ARID1A encodes an essential subunit of the mammalian SWI/SNF chromatin remodelling complex. This domain is involved in multiple cellular processes, such as DNA replication, DNA damage repair, tumour metabolism [10] and transcription [11]. ARID1A is the most frequently mutated subunit of the chromatin remodelling complex and the most commonly mutated gene in cancers. ARID1A mutations occur in approximately 7% ~ 17% of HCC [8, 12–14]. The majority of ARID1A mutations are loss-of-function mutations and thus result in ARID1A deficiency. Early research focused on the tumour suppressor role of ARID1A and ARID1A deficiency, which are associated with worse prognostic outcomes in various cancers [15, 16]. However, increasing evidence has shown that ARID1A deficiency may modulate the tumour immune system, correlating it with better therapeutic outcomes of ICBs. These observations have highlighted that ARID1A might serve as a new biomarker for immunotherapy [17–19]. In addition, ARID1A deficiency was associated with a higher TMB, more infiltrating lymphocytes, and increased PD-L1 expression in various tumours [20, 21].Moreover, mice with ARID1A-deficient ovarian cancers showed a prolonged survival rate when treated with ICBs [22]. Overall, both the clinical and preclinical evidence suggest that ARID1A deficiency might influence immune activity and synergistically enhance the effects of ICBs.

In this study, we focused on HBV-HCC, the most important prevalent subtype of HCC. We used bioinformatics approaches and preclinical experiments to evaluate the ARID1A regulatory role in the biological behaviors and immune modulation of HBV-HCC and its potential immunotherapeutic implications.

## Materials and methods

### Data

Three datasets were explored in this study. In the CHCC-HBV cohort, patients were selected as hepatitis B core antibody (HBcAb) and hepatitis B surface antigen (HBsAg) positive. A total of 150 patients were enrolled in the analysis, among which 15 patients had ARID1A deficiency, as they carried loss-of-function mutations or copy number deletions in ARID1A. The expression data were downloaded from https://www.biosion.org [7]. The mutation data were obtained from the supplementary information of the CHCC-HBV study [23]. For the TCGA-LIHC cohort, the infection status of HBV was obtained from two studies, which contained 44 and 87 HBV patients [12, 23]. A total of 108 HBV-infected patients were enrolled from the TCGA-LIHC cohort after removing duplicates and patients with mutations or missing clinical information. For the AMC cohort, only 167 patients identified to have HBV infection were included in this study [24]. All the original data can be downloaded at cBioPortal (https://www.cbioportal.org). Overall, 425 HBV-related hepatocellular carcinoma patients were enrolled in the study. The clinical information of patients included in this study can be found in Supplementary file S1.

### Tumour Mutation Burden (TMB)

Tumour mutation burden was calculated as the number of nonsynonymous mutations per targeted sequencing length. The exon length from the TCGA cohort was estimated as 38 Mb [25], while the CHCC and AMC cohorts used the Agilent SureSelect 50 Mb system to capture the exon area [7, 24].

### Gene set enrichment analysis

Based on the Molecular Signatures Database (MSigDB), gene set enrichment analysis (GSEA) was performed to correlate the CHCC-HBV cohort grouped by ARID1A mutation status to the known hallmark gene expression signatures. The normalized gene expression of the CHCC-HBV cohort was taken as the input. We followed the GSEA user’s guide using default parameters to run the software. An FDR corrected *q*-value < 0.05 was considered statistically significant.

### Immune cell infiltration level analysis

The immune infiltration level was estimated by single-sample GSEA using the Gene Set Variation Analysis (GSVA) program against the gene signatures representing immune cell populations (Supplementary file S2). In addition, the immune infiltration status of the tumour purity, immune components, and overall stromal status in the CHCC-HBV cohort was computed using ESTIMATE.

### Cell culture and CRISPR knockout

The human hepatocellular carcinoma cell lines Hep3B and SK-HEP-1 were purchased from ATCC. Cells were grown in Dulbecco’s modified Eagle medium (Gibco™; Thermo Fisher Scientific, MA, USA) supplemented with 10% foetal bovine serum (Gibco™; Thermo Fisher Scientific) and 1% penicillin‒streptomycin (Gibco™; Thermo Fisher Scientific) and maintained in a humidified incubator at 37 °C with 5% CO2. Hep3B and SK-HEP-1 cells stably expressing Cas9 were established by lentiviral transduction with Cas9 plasmids (Lenti-Cas9-2A-Blast, 73310; Addgene).

ARID1A knockout lentiviral plasmids were synthesized and purchased from GenePharma (Beijing, China). Plasmid psPAX2 (12260; Addgene), pmd2G (12259; Addgene), and ARID1A Knockout lentiviral plasmids were cotransfected into 293 T cells by PEI (Proteintech, #PR40001). First, Hep3B and SK-HEP-1 cells were infected with lentivirus containing polybrene (10 μg/ml) (TR-1003; Sigma‒Aldrich) for 24 h. Then, positive cells were selected with 100 μg/ml hygromycin B. The efficiency of ARID1A knockout was confirmed by Western blotting.

The sgRNA sequences were as follows:

- ARID1A-sgRNA1: 5’- CAGCAGAACTCTCACGACCACGG -3’ (Exon 1),
- ARID1A-sgRNA2: CCTGTTGACCATACCCGCTGGGG -3’ (Exon 3)

### siRNA-mediated gene silencing

The siRNA duplexes and negative control were synthesized and purified by GenePharma. (Beijing, China). Briefly, HepG2/2.2.15 cells were transfected with negative control siRNA using Lipofectamine™ 3000 (#L3000001; Invitrogen) according to the manufacturer’s instructions. Knockdown efficiency was tested by Western blot analysis 48 h after transfection. siRNA sequences used for ARID1A knockdown can be found in Supplementary file S3.

### Western blot

Lysis buffer (R0010–100 ml, Solarbio) was used to extract proteins from Hep3B and SK-HEP-1 cells. Cells were washed twice with ice-cold PBS, and then protein lysates were separated by 12.5% SDS‒PAGE at 120 V for 70 min (Omni-Easy™ One-Step PAGE Gel Fast Preparation Kit, PG213; EpiZyme, Shanghai, China) and transferred to PVDF membranes (IPVH00010; Merck Millipore) with transfer buffer plus 10% methanol on ice at 300 mA for 2.5 h. (Transfer Buffer (10x), PS109; EpiZyme, Shanghai, China). Subsequently, the membranes were blocked in 5% skim milk for 1 h and incubated overnight with antibodies against ARID1A (rabbit monoclonal antibody, 1:1000, HPA005456; Sigma‒Aldrich) followed by secondary antibodies (goat anti-rabbit IgG H&L (HRP), ab6721; goat anti-mouse IgG H&L (HRP), ab6789; Abcam). β-Actin (mouse monoclonal antibody, 1:2000; ab8226; Abcam) was used as the loading control. Images were visualized using chemiluminescent horseradish peroxidase substrate (WBKLS0100; Merck Millipore) on Bio-Rad Gel Doc 2000 system analysis software (Bio-Rad Laboratories, Inc., Hercules, CA).

### RNA extraction and real-time quantitative PCR

Total RNA was isolated from harvested cells using TRIzol reagent (Invitrogen, Thermo Fisher Scientific). cDNA synthesis was performed with 1 μg of total RNA using PrimeScript RT Master Mix (RR036;ATakara). In addition, quantitative reverse transcription-PCR (qRT‒PCR) was performed using TB Green® Premix Ex Taq™ II (RR820A; Takara) on a quantitative PCR machine (qTOWER3G, Analytik Jena, Jena, Germany). The primers used for qPCR are listed in Supplementary file S3.

### Cell proliferation analysis

The effects of ARID1A deficiency on HCC proliferation were assessed by using a Cell Counting Kit-8 (CCK-8) (HY-K0301; MedChemExpress) and clone formation assays. Hep3B, SK-HEP-1 wild-type cells, and knockout cells were collected and seeded in a 96-well plate (3,000 cells per well). In addition, 10 μL of CCK-8 solution was added to each well at 24 h, 48 h, 72 h, and 120 h. The optical density (OD) of each well was measured (EnSpire, PerkinElmer, CA, USA) at 450 nm 2 h after incubation. For clone formation, 2000 cells were seeded into a six-well plate and cultured for 2 weeks or more. The cells were then fixed with Paraformaldehyde Fix Solution for 30 min and stained with 0.1% crystal violet for 15 min. At least three replicate samples were analysed for all assays.

### Cell migration and invasion assay

Transwell migration and invasion assays were performed using 8-μm-pore inserts (Corning Incorporated Costar, Tewksbury, USA). For the migration assay, cells (5 × 104 cells per well) were seeded into the upper chamber directly with serum-free medium. For the invasion assay, the inserts were coated with 50 µL of Matrigel (Solarbio, Beijing, China), and 600 µL culture medium containing 15% FBS was added to both lower chambers. Migrated or invaded cells were stained with 0.1% crystal violet 24 h after culture and counted in three random fields under light microscopy in a 100 × scope.

### Immunohistochemistry

We retrospectively reviewed the data of 19 consecutive HCC patients admitted to the oncology department of Peking University International Hospital between 1 April and 30 September 2020 and performed next-generation sequencing of tumour tissues. Three patients had ARID1A mutations. Paraffin sections from HCC patients were baked at 60 °C for 2 h, deparaffinized, and hydrated with xylene and graded alcohol. Heat-based antigen retrieval was performed using citrate or EDTA-containing buffer. Hydrogen peroxide (3%) was used to block endogenous peroxidase in tissues, which were then incubated with primary antibodies against ARID1A (rabbit monoclonal antibody, 1:100, HPA005456; Sigma‒Aldrich), TIM-3 (rabbit monoclonal antibody, 1:200, ab241332; Abcam), CD8 (rabbit monoclonal antibody, 1:200, ZA-0508; Zhongshan Goldenbridge) and CD56 (rabbit monoclonal antibody, 1:200, ZM-0057; Zhongshan Goldenbridge) overnight. Furthermore, the slides were incubated with polyperoxidase-anti-mouse/rabbit IgG (PV-9000; Zhongshan Goldenbridge) for 30 min. Finally, the slides were stained with DAB and haematoxylin. For each section, three random fields were captured under light microscopy (Carl Zeiss, Primovert, NY) (100 × scope; 200 × scope) and analysed by ImageJ software.

### Flow cytometry

Flow cytometric analysis was performed to determine the effects of ARID1A deficiency on galectin-9 expression. Cells were collected and washed twice with ice-cold PBS and then incubated with Alexa Fluor® 488 anti-human Galectin-9 Antibody (348918; Biolegend) for 15 min. Finally, Galectin-9 expression was detected using FCM (FACS Calibur flow cytometer, BD Biosciences) and analysed using FlowJo software.

### Statistics

Two-sided Fisher’s exact tests and Mann‒Whitney U tests were applied to compare the two groups. Overall survival was estimated by Kaplan‒Meier analysis. R 3. 6. 1 package was used for all analyses. Preclinical experiments and data analyses were performed using GraphPad Prism v8.0. Statistical significance was determined by Student’s t test between two groups. *P* < 0.05 was considered statistically significant.

## Results

### ARID1A loss-of-function mutations and copy number deletions are correlated with ARID1A deficiency and confer worse overall survival in HBV-related HCC

**Fig. 1:**
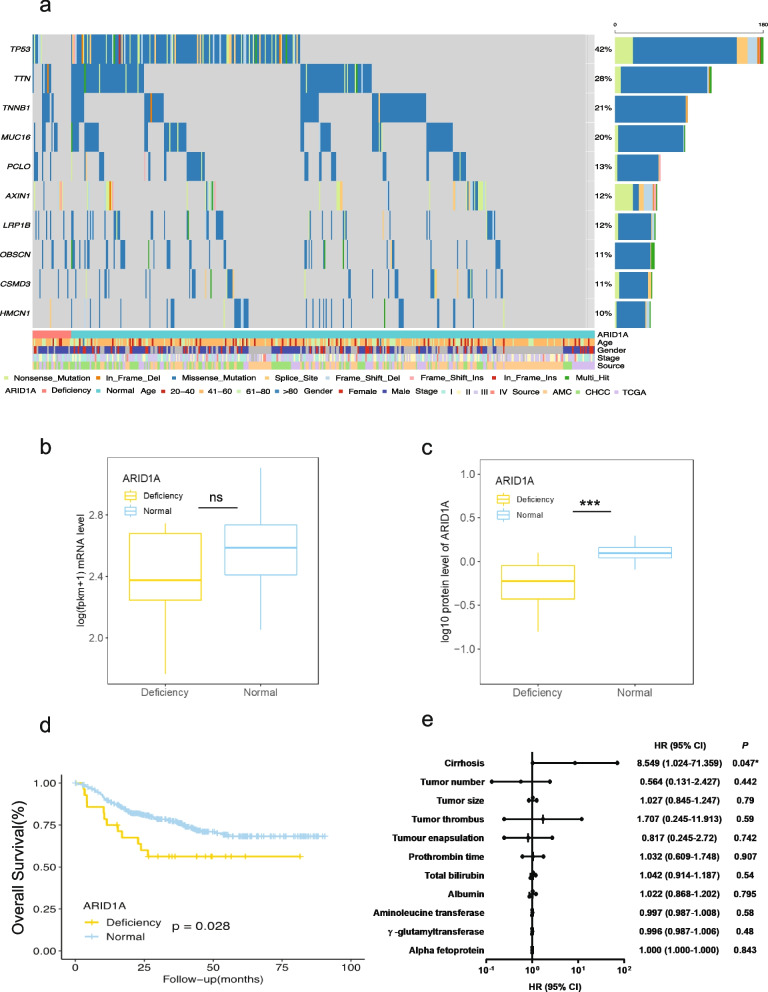
ARID1A loss-of-function mutations and copy number deletions correlated with ARID1A deficiency and were associated with worse overall survival in HBV-related HCC. **a** Mutation status of the top 20 mutated genes, including ARID1A, is presented in all three cohorts. Patients were grouped according to the mutations in ARID1A. The histogram on top shows the number of nonsynonymous mutations in each patient. The histogram on the right shows the number of mutations in each gene. Clinical characteristics are plotted at the bottom. **b**, **c** ARID1A mRNA showed no significant difference between the two groups. At the same time, the protein expression of ARID1A was significantly higher in the normal functioning group; expression and protein level are log-transformed. **d**, **e** HBV-related HCC patients with ARID1A deficiency were associated with cirrhosis and had worse overall survival. (* *P* < 0.05, ** *P* < 0.01, *** *P* < 0.001)

We integrated the whole-exome sequencing data from three cohorts, including TCGA-LIHC, AMC, and CHCC-HBV. In total, 425 HBV-related hepatocellular carcinoma patients with a median age of 53 years (range 20–83) were included in the study (Supplementary file S1). Approximately 10% of patients carried mutations in ARID1A, and the most frequently mutated gene was TP53 (Fig. 1a). We primarily focused on ARID1A mutations; only samples with frameshift mutations, splice site mutations, nonsense mutations, or copy number (CN) deletions were exclusively considered for the ARID1A deficient group. In contrast, the rest of the samples were defined as the ARID1A normal functioning group. Eventually, a total of 29 (6.8%) samples were classified as the ARID1A deficient group.

To verify the accuracy of the methods, we first analysed the mRNA and protein levels of ARID1A in the CHCC-HBV cohort between the deficient and normal functioning groups. A total of 150 patients, with 15 (10%) from the ARID1A deficient group, were divided into two groups following the previously described standards. There was no significant difference in mRNA expression between the two groups (Fig. 1b). In contrast, the protein expression of ARID1A was significantly higher in the normal functioning group (Fig. 1c). The findings confirmed that frameshift mutation, splice site mutation, nonsense mutation, and copy number deletions resulted in ARID1A deficiency. In addition, the mRNA expression of ARID1A did not correlate with the protein expression, and according to the Human Protein Atlas database, low expression of ARID1A mRNA was associated with better survival because the low expression of mRNA does not necessarily represent ARID1A deficiency at the protein level. Therefore, we confirmed that frameshift mutations, splice site mutations, nonsense mutations, and copy number deletions led to decreased protein levels and ARID1A deficiency in HBV-related HCC. In addition, the clinical implication of ARID1A deficiency in HBV-related HCC was assessed using a Kaplan–Meier analysis of overall survival (OS) from the integrated dataset. The OS of the ARID1A deficient group was significantly worse than that of the normal functioning group (Fig. 1d). This observation concurs with previous studies [26], indicating that ARID1A may serve as a tumour suppressor gene. Further analysis revealed that the ARID1A deficient group was associated with cirrhosis (Fig. 1e), which is the main risk factor for HCC initiation.

### ARID1A deficiency promotes the proliferation and metastasis of HCC in vitro

**Fig. 2:**
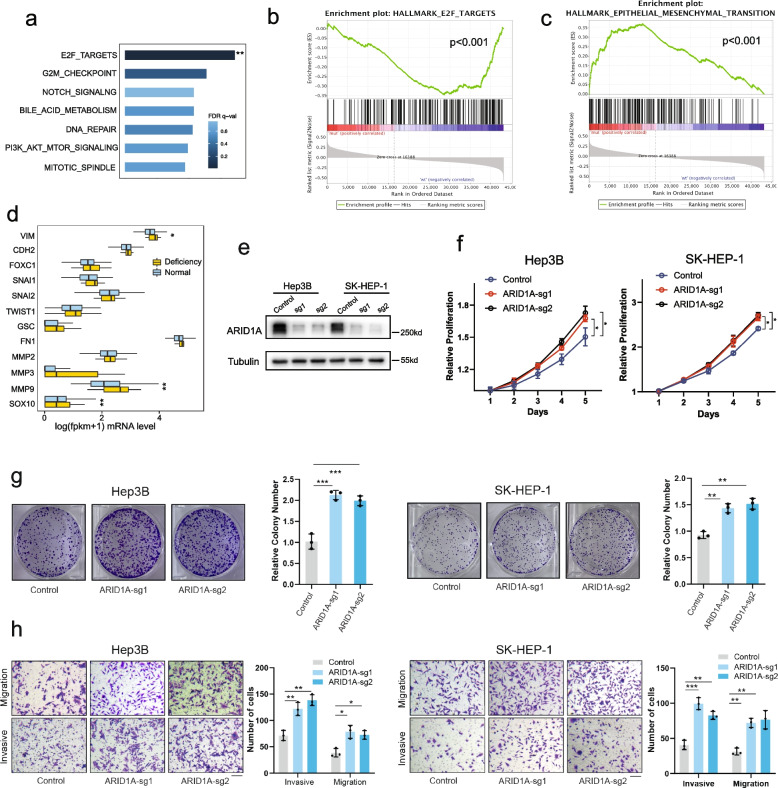
Bioinformatic analysis and in vitro experiments revealed that ARID1A deficiency promotes HCC cell proliferation, migration, and invasion in vitro. **a**, **b** GSEA identified that the cell cycle-related E2F pathway was more enriched in the ARID1A normal group. **c** GSEA identified that the epithelial-mesenchymal transition (EMT) pathway was highly enriched in the ARID1A-deficient group compared with the ARID1A normal functioning group. **d** The ARID1A-deficient group had higher mesenchymal marker gene expression. **e** ARID1A CRISPR knockout in Hep3B and SK-HEP-1 cell lines was confirmed by Western blotting. **f** CCK-8 assays showing cell viability of ARID1A in WT and ARID1A knockout SK-Hep-1 and Hep3B cells (*n* = 6). **g** Clone formation of ARID1A knockout Hep3B and SK-HEP-1 cells compared with their controls (*n* = 3). **h** Image results of migration and invasion assays of HCC cells between ARID1A knockout and control cells (*n* = 3). (* *P* < 0.05, ** *P* < 0.01, *** *P* < 0.001), Scale bar = 100 μm

To gain biological insight into pathways affected by ARID1A deficiency, we performed gene set enrichment analysis (GSEA). The cell cycle-related E2F pathway, consisting of genes necessary for progression through the S phase of the cell cycle, was highly enriched (Fig. 2a, b). Another pathway enriched in the ARID1A deficient group was the epithelial-mesenchymal transition (EMT) pathway, which is relevant to metastasis, invasion, and more aggressive performance (Fig. 2c). We further explored the gene markers related to cells in epithelial or mesenchymal states. Several mesenchymal markers had increased expression of matrix metalloproteinase 3 (MMP3), matrix metalloproteinase 9 (MMP9), and vimentin (VIM) in the ARID1A deficient group (Fig. 2d).

Upregulation of mesenchymal markers indicated that HCC cells with ARID1A deficiency were stimulated to undergo the EMT process, which eventually led to metastasis. The results of bioinformatics analysis were supported by in vitro experiments. We performed ARID1A CRISPR knockout in the human HCC cell line SK-HEP-1 and the Hep3B cell line to investigate the function of ARID1A in vitro. Western blotting confirmed the efficacy of CRISPR knockout (Fig. 2e). The decreased expression of ARID1A promoted proliferation (Fig. 2f, g), migration, and invasion in vitro (Fig. 2h).

### ARID1A deficiency correlated with an increased tumour mutation burden and a higher mutation rate in DDR pathways

**Fig. 3:**
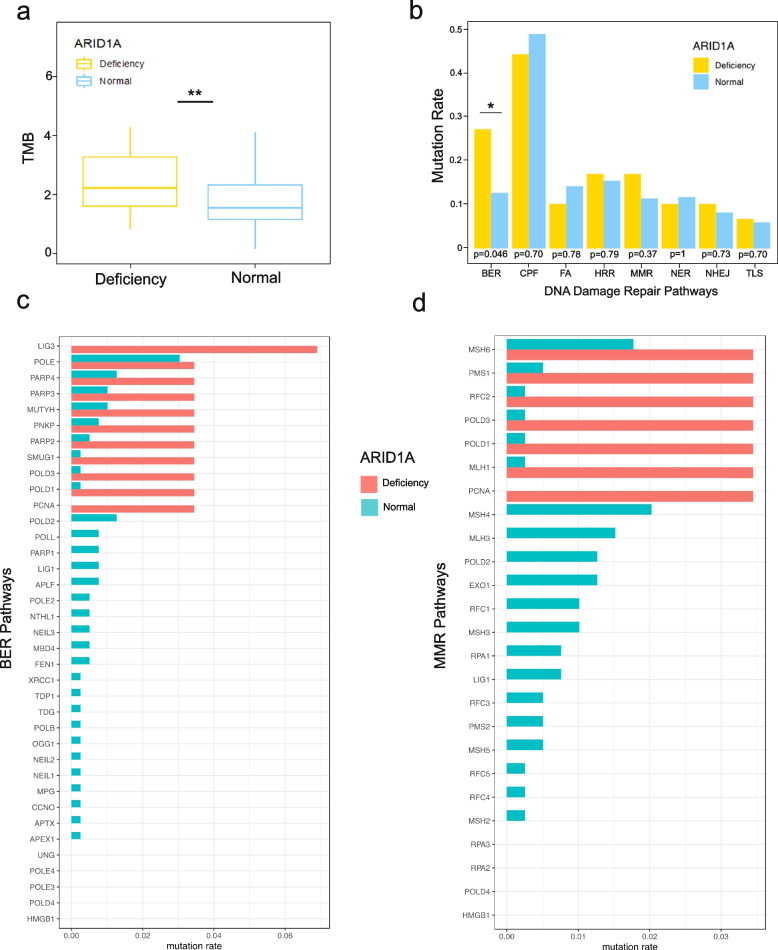
ARID1A deficiency correlated with increased tumour mutation burden and higher mutation rate in DDR pathways. **a** ARID1A-deficient HBV-HCC had a significantly higher tumour mutational burden. **b** The difference in the mutation rate of DNA damage repair pathways between the two groups. **c**, **d** ARID1A deficiency significantly coexisted with mutations in base-excision repair (BER) genes and mismatch repair (MMR) genes. (* *P* < 0.05, ** *P* < 0.01, *** *P* < 0.001)

To investigate the immunomodulatory role of ARID1A in HBV-HCC, we calculated the TMB, which was measured by the quantity of somatic mutations per Mb in the tumour genome. Then, the data from the three cohorts were normalized based on the length of the sequenced target region. Compared with the ARID1A normal function groups, the ARID1A deficient group exhibited a higher TMB level (Fig. 3a). This observation indicates that ARID1A deficiency was associated with the genomic instability of HBV-HCC. Moreover, the finding was consistent with our previous study on HCC and studies of other tumours.

Previous studies have shown that ARID1A deficiency might impair the DNA mismatch repair (MMR) pathway [19], implying the underlying mechanism of increased mutagenesis. Therefore, we investigated the mutational rate of the eight DNA damage repair (DDR) pathways. We found that ARID1A deficiency was more likely to display dysregulation in these pathways, out of which the base excision repair (BER) pathway exhibited a significantly higher mutational frequency in the ARID1A deficient group (Fig. 3b, c). Although not statistically significant, the same trend was observed in the MMR pathway. In addition, mutations in the MMR pathway occurred only in certain genes, including MSH6, MLH1, and PMS1 (Fig. 3d). Overall, an alternative mechanism for the high mutational rate in DDR pathways might impair the DNA repair function and result in genomic instability in HBV-HCC with ARID1A deficiency.

### ARID1A deficiency is correlated with elevated immune activity in tumour tissues

**Fig. 4:**
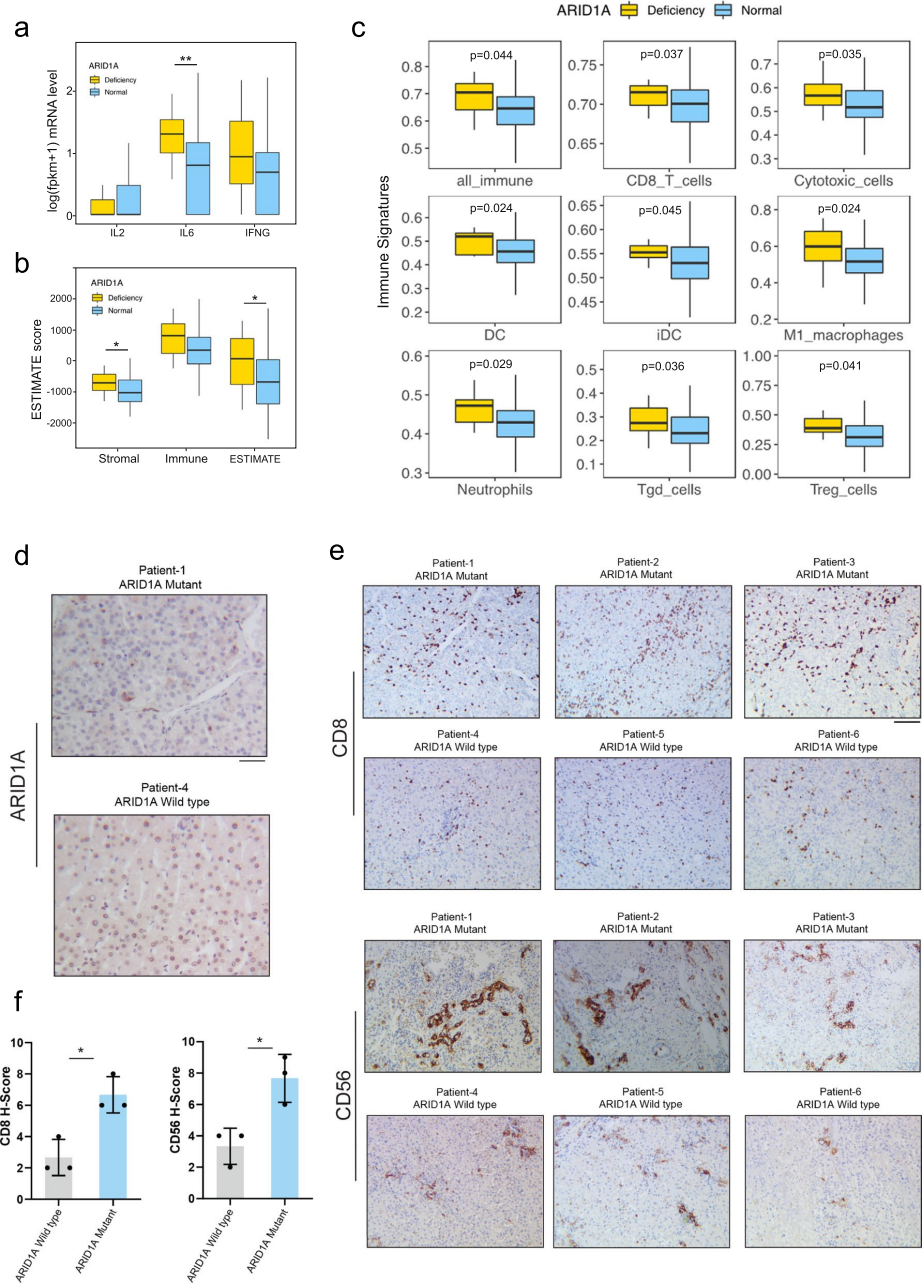
ARID1A deficiency correlated with elevated immune activity in tumour tissues. **a** The different IL2, IL6, and IFN γ expression levels between the two groups. **b** Immune infiltration score by ESTIMATE. **c** The difference in lymphocyte infiltration level by ssGSEA in the two groups. **d**-**f** Representative images, quantitation of immunostaining, and immunohistochemical staining for ARID1A, CD8, and CD56 expression in HCC patients (ARID1A magnification × 200; CD8 and CD56 magnification × 100). Scale bar = 100 μm or 50 μm. (* *P* < 0.05, ** *P* < 0.01, *** *P* < 0.001)

Gene set enrichment analysis revealed that ARID1A deficiency was positively correlated with active immune signatures, including the interleukin 6 (IL6) and IFN γ pathways (Fig. 4a). Notably, there was a significantly higher mRNA expression level of IL6 in the ARID1A-deficient group for the IL6 pathway. Based on the mRNA transcription data, we next estimated the activity of stromal and immune cells present in tumour tissues. The ARID1A-deficient group had higher ESTIMATE scores for stromal and immune cells (Fig. 4b). Further investigation of the immune cells infiltrating tumour tissues revealed multiple enriched immune signatures in the ARID1A-deficient group (Fig. 4c). CD8 + T cells, which are important for the immune surveillance of tumours, were also significantly enriched along with other types of cells, including neutrophils, M1 macrophages, and cytotoxic cells. We further confirmed elevated immune activity in ARID1A-deficient HCC. HCC specimens from 6 patients were stained for ARID1A, CD8, and CD56 and examined by immunohistochemistry (IHC). The patient-1, 2, and 3 had p.G1500W mutation, p.G108Efs*7 mutation, and p.S1985Y mutation in ARID1A, respectively, and can be classified as the ARID1A deficiency (Fig. 4d), wide type ARID1A (patient-4,5,6) in three HCC patients as control. IHC staining of these tumours additionally revealed that the loss of ARID1A significantly upregulated CD8 and CD56 infiltrating levels in these tumours (Fig. 4e, f). These findings were consistent with the bioinformatic analysis. Overall, ARID1A deficiency was associated with increased immune activity in tumour tissues.

### ARID1A deficiency was associated with the elevated expression of immune checkpoints

**Fig. 5:**
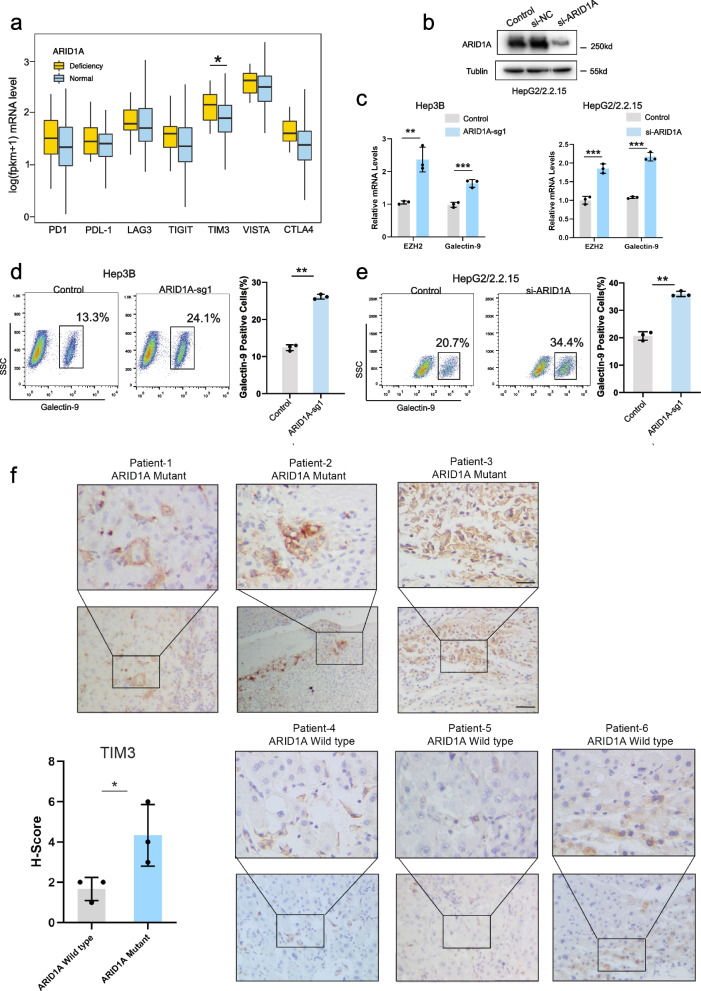
ARID1A deficiency was associated with elevated immune checkpoint expression and upregulated Galectin-9 expression. **a** ARID1A deficiency has significantly higher expression levels of the immune checkpoint. **b** ARID1A knockdown by siRNA in HepG2/2.2.15 cell line was confirmed by Western blotting. **c** EZH2 and galectin-9 mRNA levels were measured after ARID1A knockout or knockdown (*n* = 3). **d**, **e** Representative flow cytometric analysis showing galectin-9-positive cells in Hep3B and HepG2/2.215 cells. **f** Immunohistochemical staining for TIM3 expression within patients carry ARID1A mutation or not. Scale bar = 100 μm or 50 μm. (* *P* < 0.05, ** *P* < 0.01, *** *P* < 0.001)

ARID1A deficiency is associated with elevated PD-L1 expression in various cancers [27–29]. Thus, we performed an analysis to explore the relationship between ARID1A deficiency and immune checkpoint protein expression. Our analysis revealed no significant difference in PD-L1 expression between the ARID1A deficiency and normal functioning groups. Nevertheless, the expression of TIM-3, which is another inhibitory immune checkpoint, was significantly higher in the deficient group (*p* = 0.004) (Fig. 5a). TIM-3, a coinhibitory receptor in the immune response, interacts with its ligand galectin-9. This interaction results in CD8-positive T-cell exhaustion and suppressed immune responses, especially in chronic viral infections [19]. Moreover, recent studies have shown that IFN-γ could stimulate galectin-9 expression in tumour and immune cells via EZH2 [30, 31]. Our bioinformatic analysis revealed an active IFN-γ signalling pathway in the ARID1A-deficient group. Therefore, we assessed whether the expression of galectin-9 could be upregulated in ARID1A knockout HCC cells. Surprisingly, increased galectin-9 expression was observed in the ARID1A knockout Hep-3B cell line and ARID1A knockdown HepG2/2.2.15 cell line (Fig. 5b-e), whereas the increase was not observed in the SK-HEP-1 cell line. This observation implied that the activity of the TIM-3/Galectin-9 pathway was upregulated only in HBV-HCC. Furthermore, IHC staining for TIM-3 and ARID1A in human HCC samples confirmed a strong negative correlation between the levels of these two proteins (Fig. 5f). Thus, the TIM-3-Galectin-9 interaction might play an essential role in immune suppression in HBV-HCC with ARID1A deficiency. Furthermore, this interaction might be an underlying mechanism for primary and secondary drug resistance to ICB treatment in HBV-HCC. In contrast, the combined inhibition of TIM-3 with current therapeutic approaches might achieve a superior response in HBV-HCC with ARID1A deficiency.

## Discussion

Exploring predictive biomarkers and therapeutic strategies for ICBs has become an urgent need in clinical practice. Increasing evidence indicates that genomic alterations inside tumour cells determine their phenotypic performance and the surrounding microenvironment. Therefore, such genes might be explored as predictive biomarkers for prognostic and therapeutic outcomes. ARID1A is a subunit of the SWI/SNF family and is involved in various cellular processes. The effects of ARID1A deficiency potentiate therapeutic antitumour immunity and thus might serve as a predictive biomarker for ICB therapy outcomes. The plausibility of ARID1A as a biomarker was confirmed by Jiang et al. through pan-cancer analysis using an online database [18]. As ARID1A is one of the most frequently mutated genes in HCC and ICBs, it has become one of the main treatment options for HCC. Therefore, in the present study, we explored the immune-modulating role of ARID1A deficiency in HBV-HCC and its potential immunotherapeutic implications.

Consistent with previous reports [26], we confirmed the tumour-suppressive effect of ARID1A using bioinformatics approaches and preclinical experiments. Furthermore, ARID1A deficiency promoted growth, migration, and invasion. These results imply a more aggressive biological feature and worse overall survival of HBV-HCC with ARID1A deficiency.

Previously, we conducted genome-wide next-generation sequencing of 81 HCC tissue samples and found that ARID1A alterations were significantly correlated with a higher tumour mutation burden in HCC [32]. In this study, the TMB of HBV-HCC with ARID1A deficiency was significantly higher than the TMB of HCC without ARID1A deficiency. Apart from our study, ARID1A deficiency was observed to induce a similar MMR phenotype, correlating with microsatellite instability-high (MSI-H) and higher TMB (TMB-H) in ovarian cancer [22]. In gastric cancer, ARID1A deficiency was correlated with dMMR and increased expression of PD-L1 [33]. The ARID1A deficiency resulted in its inability to recruit MSH2 to chromatin during DNA replication, compromising the MMR and increasing mutagenesis [22]. In addition, ARID1A has synthetic lethal effects with PARP inhibitors and ATR inhibitors. These effects were synthetically lethal with mutations in the DDR pathway, such as BRCA1/2, indicating a tightly intertwined relationship between DDR and ARID1A deficiency [34, 35]. For the first time, we reported that HBV-HCC with ARID1A deficiency had a higher mutation rate in the DDR pathway. This might be an alternative mechanism of DDR impairment and TMB-H in ARID1A deficiency in HBV-HCC.

Furthermore, the present study revealed that HBV-HCC with ARID1A deficiency had a higher level of immune cell infiltration in tumour tissues, which is characteristic of the so-called “hot tumour” and is more prone to ICBs [33]. In addition, CD8 + cytotoxic T lymphocytes, the main executors of antitumour immunity, and other kinds of immune cell enrichment, such as natural killer cells (NK cells) and dendritic cells (DCs), also accumulated in the ARID1A-deficient group. These cells are critical for antitumour effects through antigen presentation, cytokine secretion, lymphocyte chemotaxis, etc. [36, 37]. In addition, GSEA showed multiple enriched immune-related pathways, including the IL6 and IFN-γ signalling pathways, in ARID1A-deficient HBV-HCC, which are important for antitumour activity conducted by immune cells, especially T lymphocytes. These results suggest that ARID1A deficiency is associated with elevated therapeutic immunity in HBV-HCC. Therefore, consistent with studies of other tumours, it might serve as a predictive biomarker for ICB treatment.

T-cell dysfunction is a major mechanism for immune escape. Hence, most current immunotherapeutic strategies act on reactive T cells to exert antitumour effects. However, in addition to the expression of several immune checkpoints, including PD-1, PD-L1, and CTLA4, TIM-3, another inhibitory immune checkpoint expressed in T cells has been investigated to induce tumour infiltrating CD8 + T lymphocyte exhaustion in HCC [38]. For the first time, we reported that ARID1A deficiency was correlated with the increased expression of TIM-3. In contrast, the expression of Galectin-9, which is the ligand of TIM-3, could also be upregulated in ARID1A knockout Hep-3B or knockdown HepG2/2.2.15 cell line but not in the SK- HEP -1 cell line. Hep-3B and HepG2/2.2.15 is the HBV-positive HCC cell line, while SK-HEP-1 is not. This observation implied that the activity of the TIM-3/Galectin-9 pathway was upregulated only in HBV-HCC. Thus, the combined blockade of PD-1/PD-L1 with TIM-3 might be a better option to revitalize the antitumour activity of infiltrating T lymphocytes in HBV-HCC with ARID1A deficiency. Furthermore, NSCLC patients whose disease progressed after treatment with PD-1 inhibitors showed higher expression of TIM-3. Importantly, the TIM-3 antibody could overcome resistance to PD-1 blockade in mouse models of lung cancer [39]. Therefore, the increased activity of the TIM-3/Galectin-9 pathway might be one of the mechanisms for primary or secondary drug resistance during ICB therapy. Therefore, combining inhibition of both pathways might be a promising strategy to overcome the drug resistance of PD-1/PD-L1 inhibitor monotherapy in HBV-HCC patients with ARID1A deficiency.

For predicting the therapeutic efficacy of ICBs, PD-L1 expression, tumour-infiltrating lymphocytes, TMB, dMMR, and MSI-H are the most commonly explored biomarkers [36]. However, it has several limitations in clinical practice. First, there is spatiotemporal heterogeneity in PD-L expression. The immunohistochemical staining of PD-L1 might be inaccurate; the testing range and optimal threshold for defining the positivity of TMB are still unknown; next-generation sequencing (NGS) for determining the TMB and MSI-H is expensive and time-consuming. Second, the predictive value of these markers is very limited in HCC. Furthermore, dMMR expression was observed in only 2–3% of HCC [40], although the positive expression of PD-L1 was as high as 42–75% in HCC. Moreover, PD-L1 positivity (in terms of TPS) and the therapeutic efficacy of PD-1 antibodies were not significantly correlated in either the KEYNOTE 240 or CheckMate-040 study [5]. There is an urgent need to explore biomarkers applicable to clinical use in HCC. Considering the malignant biological features of the tumour, including the immune escape capability of tumour cells due to its genetic alterations [37], it is logical to hypothesize that genetic alterations within tumour cells might be the vital underlying factors that shape the tumour immune fate by driving specific immune-related pathways. For example, WNT/β-catenin pathway activation could lead to ICB therapy resistance in melanoma by inducing the absence of tumour-infiltrating lymphocytes and T-cell exhaustion [38]. In addition, HCC with activating mutations in the WNT/β-catenin pathway also demonstrated worse median progression-free survival (mPFS) and median overall survival (mOS) for patients treated with ICBs [39]. In our study, ARID1A deficiency greatly impacted multiple aspects of immune signatures in HBV-HCC. Therefore, ARID1A deficiency may serve as a stand-alone or combined predictive marker for ICB therapy in HBC-HCC.

Our study had several limitations. First, most results were based on bioinformatics approaches and in vitro experiments. In vivo experiments and data from clinical practice are required to further validate our study's results. Second, the underlying mechanisms of ARID1A interacting with this immune signature are still unknown and need to be investigated further.

In conclusion, we confirmed the tumour-suppressive effect of ARID1A in HCC. For the first time, we have shown that ARID1A deficiency was correlated with more active immune signatures and higher expression of immune checkpoints in HBV-HCC. Furthermore, our study suggests that ARID1A deficiency might be a promising predictive biomarker for assessing the therapeutic outcomes of ICBs in HBV-HCC. The combination of TIM3 blockades and current therapeutic approaches might help achieve a superior response.

### Supplementary Information

**Additional file 1: Supplementary File S1.** Clinical information of 425 patients with HBV infection.

**Additional file 2: Supplementary File S2.** ssGSEA score of immune signatures.

**Additional file 3. Supplementary File S3. **Oligonucleotides used in this study.

**Additional file 4.**

## Data Availability

All data generated or material during this study are included in this article and its Supplementary files.
