# Supplementary material for: ARID1A deficiency promotes progression and potentiates therapeutic antitumour immunity in hepatitis B virus-related hepatocellular carcinoma
Source: BMC Gastroenterol. 2024 Jan 2;24:11. doi: 10.1186/s12876-023-03059-w (PMC10759659; doi:10.1186/s12876-023-03059-w)
Supplement: Supplementary file 1 — Additional file 1: Supplementary File S1. Clinical information of 425 patients with HBV infection. [file 12876_2023_3059_MOESM1_ESM.pdf]

Supplementary File S1. Clinical information of 425 patients with HBV infection

Patient	ARID1A	Age
H050226	Normal	41-60
H050267	Normal	41-60
H050313	Normal	41-60
H050329	Normal	41-60
H050350	Normal	41-60
H050401	Normal	41-60
H050420	Normal	41-60
H050452	Normal	61-80
H050510	Normal	61-80
H050566	Normal	20-40
H050572	Normal	41-60
H050604	Normal	41-60
H060030	Normal	41-60
H060098	Deficiency	41-60
H060115	Normal	41-60
H060236	Normal	41-60
H060264	Normal	61-80
H060362	Normal	41-60
H060509	Normal	61-80
H060555	Normal	61-80
H060607	Normal	61-80
H060616	Normal	41-60
H060617	Normal	41-60
H060664	Normal	41-60
H060670	Deficiency	41-60
H060674	Normal	41-60
H060690	Normal	41-60
H060795	Normal	41-60
H060859	Normal	41-60
H061039	Normal	41-60
H061142	Normal	41-60
H061840	Normal	41-60
H062024	Normal	20-40
H070487	Normal	41-60
H070599	Normal	41-60
H071380	Normal	41-60
H071591	Normal	41-60
H071859	Normal	20-40
H071899	Normal	41-60
H071904	Normal	41-60
H071941	Normal	41-60
H072012	Normal	61-80
H072032	Normal	20-40
H072059	Normal	41-60
H072103	Normal	41-60
H072441	Normal	20-40
H072447	Normal	41-60
H072511	Normal	61-80
H072525	Normal	20-40
H072543	Normal	41-60
H072601	Normal	41-60
H072623	Normal	41-60
H072749	Normal	61-80
H072777	Normal	41-60
H072813	Normal	41-60
H072820	Normal	41-60
H072849	Normal	41-60
H072896	Deficiency	41-60
H072926	Normal	41-60
H072932	Normal	41-60
H072961	Normal	20-40
H072992	Normal	41-60

H072999	Normal	41-60
H080015	Normal	41-60
H080025	Normal	41-60
H080037	Normal	61-80
H080061	Normal	41-60
H080074	Normal	41-60
H080174	Normal	41-60
H080196	Normal	41-60
H080204	Normal	41-60
H080217	Normal	41-60
H080486	Normal	41-60
H080503	Normal	20-40
H080530	Normal	41-60
H080540	Normal	41-60
H080750	Normal	41-60
H080774	Normal	41-60
H080782	Normal	41-60
H080854	Normal	41-60
H080895	Deficiency	41-60
H080931	Normal	61-80
H080971	Normal	41-60
H081003	Normal	61-80
H081048	Normal	61-80
H081559	Normal	41-60
H090285	Normal	41-60
H090528	Normal	41-60
H090798	Normal	61-80
H090866	Normal	41-60
H090901	Normal	61-80
H091225	Normal	41-60
H091269	Normal	61-80
H091309	Normal	61-80
H091449	Normal	41-60
H091469	Normal	41-60
H091547	Normal	41-60
H091652	Normal	41-60
H091665	Normal	61-80
H091689	Normal	41-60
H091730	Normal	41-60
H091794	Normal	41-60
H091831	Normal	41-60
H091867	Normal	61-80
H091869	Normal	61-80
H091908	Normal	41-60
H091920	Normal	41-60
H091972	Normal	41-60
H092093	Normal	41-60
H092293	Normal	41-60
H092452	Normal	41-60
H092610	Normal	41-60
H092773	Normal	41-60
H092789	Normal	41-60
H092933	Normal	41-60
H093001	Normal	20-40
H093002	Normal	41-60
H093030	Normal	20-40
H093032	Normal	61-80
H093134	Normal	41-60
H093428	Normal	41-60
H093471	Normal	41-60
H093528	Normal	61-80
H093609	Normal	41-60
H093624	Normal	61-80
H093650	Normal	41-60

H093770	Normal	61-80
H093843	Normal	41-60
H093867	Normal	41-60
H093904	Normal	61-80
H104025	Normal	41-60
H104044	Normal	41-60
H104351	Normal	20-40
H104362	Normal	41-60
H104490	Deficiency	61-80
H104529	Normal	61-80
H104572	Normal	41-60
H104656	Normal	41-60
H104773	Normal	41-60
H105140	Normal	41-60
H110058	Normal	41-60
H110061	Normal	61-80
H110104	Normal	41-60
H110134	Normal	41-60
H110146	Normal	41-60
H110261	Normal	41-60
H110362	Normal	41-60
H110379	Normal	61-80
H110451	Normal	41-60
H110467	Normal	61-80
H110669	Normal	20-40
H110702	Normal	41-60
H110772	Normal	41-60
H110773	Normal	61-80
H110795	Normal	41-60
H110908	Normal	41-60
H111183	Normal	61-80
H111362	Normal	41-60
H111720	Normal	41-60
H111889	Normal	41-60
H112288	Normal	61-80
H112290	Normal	61-80
H112345	Normal	41-60
H112352	Normal	61-80
H112562	Normal	61-80
H112665	Normal	41-60
H112971	Normal	41-60
T1013	Normal	20-40
T1015	Normal	41-60
T1021	Normal	41-60
T1025	Normal	61-80
T1027	Normal	20-40
T1031	Normal	41-60
T1041	Normal	41-60
T1043	Normal	41-60
T1045	Normal	61-80
T112	Normal	61-80
T113	Deficiency	41-60
T127	Normal	61-80
T141	Normal	>80
T187	Deficiency	41-60
T191	Deficiency	41-60
T195	Normal	20-40
T211	Normal	41-60
T217	Normal	61-80
T221	Normal	41-60
T223	Normal	61-80
T227	Normal	61-80
T231	Normal	41-60
T257	Normal	41-60

T261	Normal	61-80
T267	Normal	61-80
T271	Normal	20-40
T277	Normal	61-80
T283	Normal	61-80
T285	Normal	61-80
T311	Normal	41-60
T313	Normal	41-60
T327	Normal	41-60
T331	Normal	41-60
T341	Normal	61-80
T343	Normal	41-60
T351	Normal	20-40
T353	Normal	41-60
T355	Normal	20-40
T357	Normal	41-60
T361	Normal	41-60
T363	Normal	41-60
T365	Normal	61-80
T367	Normal	41-60
T375	Normal	41-60
T383	Normal	61-80
T385	Deficiency	61-80
T387	Normal	41-60
T391	Normal	61-80
T393	Deficiency	41-60
T395	Normal	20-40
T411	Normal	20-40
T413	Deficiency	41-60
T415	Normal	61-80
T421	Normal	41-60
T423	Normal	41-60
T425	Normal	61-80
T427	Normal	41-60
T431	Normal	41-60
T433	Normal	41-60
T435	Normal	41-60
T443	Normal	41-60
T445	Normal	41-60
T451	Deficiency	61-80
T455	Normal	41-60
T461	Normal	61-80
T463	Normal	41-60
T465	Normal	61-80
T467	Normal	41-60
T471	Normal	41-60
T473	Normal	41-60
T477	Normal	61-80
T481	Normal	41-60
T483	Normal	41-60
T487	Normal	41-60
T491	Normal	41-60
T493	Normal	61-80
T497	Normal	20-40
T513	Normal	41-60
T515	Normal	41-60
T517	Normal	41-60
T523	Normal	61-80
T525	Normal	20-40
T527	Normal	41-60
T533	Normal	20-40
T537	Normal	61-80
T545	Normal	41-60
T553	Normal	20-40

T557	Normal	41-60
T563	Normal	61-80
T567	Normal	41-60
T571	Normal	20-40
T573	Deficiency	41-60
T615	Normal	61-80
T617	Normal	41-60
T627	Normal	41-60
T635	Normal	61-80
T641	Normal	41-60
T647	Normal	20-40
T661	Normal	41-60
T663	Normal	41-60
T665	Deficiency	41-60
T671	Normal	41-60
T685	Normal	41-60
T695	Normal	61-80
T713	Normal	41-60
T715	Deficiency	41-60
T721	Normal	61-80
T724	Normal	>80
T727	Normal	61-80
T737	Normal	41-60
T741	Normal	41-60
T743	Normal	20-40
T745	Normal	41-60
T755	Deficiency	61-80
T777	Normal	20-40
T785	Normal	41-60
T813	Normal	41-60
T815	Normal	41-60
T817	Normal	41-60
T823	Normal	41-60
T851	Deficiency	61-80
T857	Normal	61-80
T861	Normal	61-80
T863	Normal	61-80
T865	Normal	41-60
T867	Normal	61-80
T873	Deficiency	41-60
T877	Normal	20-40
T881	Normal	41-60
T883	Normal	41-60
T911	Normal	61-80
T913	Normal	41-60
T915	Normal	41-60
T917	Normal	61-80
T921	Normal	41-60
T923	Deficiency	41-60
T925	Normal	61-80
T937	Normal	41-60
T943	Normal	41-60
T951	Normal	41-60
T953	Normal	61-80
T955	Normal	41-60
T957	Normal	41-60
T963	Normal	41-60
T965	Normal	61-80
T967	Normal	41-60
T975	Deficiency	20-40
T977	Normal	41-60
T981	Normal	61-80
T983	Normal	20-40
TCGA-BC-A10W-01	Deficiency	41-60

TCGA-BW-A5NP-01	Normal	20-40
TCGA-CC-5258-01	Deficiency	41-60
TCGA-CC-5259-01	Normal	41-60
TCGA-CC-5262-01	Deficiency	61-80
TCGA-CC-5263-01	Normal	20-40
TCGA-CC-5264-01	Normal	61-80
TCGA-CC-A1HT-01	Normal	41-60
TCGA-CC-A3M9-01	Normal	41-60
TCGA-CC-A3MB-01	Normal	20-40
TCGA-CC-A3MC-01	Normal	41-60
TCGA-CC-A5UC-01	Normal	61-80
TCGA-CC-A5UD-01	Normal	41-60
TCGA-CC-A5UE-01	Normal	41-60
TCGA-CC-A7IG-01	Normal	41-60
TCGA-CC-A7II-01	Normal	41-60
TCGA-CC-A7IK-01	Normal	41-60
TCGA-CC-A7IL-01	Normal	61-80
TCGA-CC-A9FU-01	Normal	41-60
TCGA-DD-A116-01	Normal	61-80
TCGA-DD-A119-01	Normal	20-40
TCGA-DD-A11A-01	Normal	61-80
TCGA-DD-A1EH-01	Deficiency	20-40
TCGA-DD-A1EI-01	Normal	41-60
TCGA-DD-A1EL-01	Normal	20-40
TCGA-DD-A3A3-01	Normal	41-60
TCGA-DD-A4NK-01	Normal	61-80
TCGA-DD-A4NQ-01	Normal	41-60
TCGA-DD-AAC8-01	Normal	61-80
TCGA-DD-AAC9-01	Normal	41-60
TCGA-DD-AACA-01	Normal	61-80
TCGA-DD-AACB-01	Normal	61-80
TCGA-DD-AACC-01	Normal	61-80
TCGA-DD-AACD-01	Normal	41-60
TCGA-DD-AACE-01	Normal	61-80
TCGA-DD-AACG-01	Deficiency	41-60
TCGA-DD-AACH-01	Normal	61-80
TCGA-DD-AACK-01	Deficiency	61-80
TCGA-DD-AACM-01	Normal	41-60
TCGA-DD-AACN-01	Normal	20-40
TCGA-DD-AACO-01	Normal	20-40
TCGA-DD-AACQ-01	Normal	41-60
TCGA-DD-AACS-01	Normal	20-40
TCGA-DD-AACT-01	Normal	61-80
TCGA-DD-AACU-01	Normal	41-60
TCGA-DD-AACY-01	Normal	61-80
TCGA-DD-AAD0-01	Normal	61-80
TCGA-DD-AAD2-01	Normal	61-80
TCGA-DD-AAD6-01	Normal	61-80
TCGA-DD-AADA-01	Normal	61-80
TCGA-DD-AADB-01	Normal	41-60
TCGA-DD-AADC-01	Normal	41-60
TCGA-DD-AADD-01	Normal	41-60
TCGA-DD-AADE-01	Normal	41-60
TCGA-DD-AADF-01	Normal	61-80
TCGA-DD-AADI-01	Normal	41-60
TCGA-DD-AADK-01	Normal	61-80
TCGA-DD-AADP-01	Deficiency	41-60
TCGA-DD-AADW-01	Normal	41-60
TCGA-DD-AADY-01	Normal	41-60
TCGA-DD-AAE0-01	Normal	41-60
TCGA-DD-AAE1-01	Normal	41-60
TCGA-DD-AAE2-01	Normal	41-60
TCGA-DD-AAE4-01	Normal	41-60
TCGA-DD-AAE8-01	Normal	41-60

TCGA-DD-AAEE-01	Normal	41-60
TCGA-DD-AAEI-01	Normal	61-80
TCGA-DD-AAEK-01	Normal	41-60
TCGA-DD-AAVP-01	Normal	41-60
TCGA-DD-AAVQ-01	Normal	20-40
TCGA-DD-AAVR-01	Normal	41-60
TCGA-DD-AAVS-01	Normal	41-60
TCGA-DD-AAVU-01	Normal	41-60
TCGA-DD-AAVV-01	Normal	41-60
TCGA-DD-AAVW-01	Normal	20-40
TCGA-DD-AAVX-01	Deficiency	20-40
TCGA-DD-AAVZ-01	Normal	20-40
TCGA-DD-AAW0-01	Normal	41-60
TCGA-ED-A459-01	Normal	41-60
TCGA-ED-A4XI-01	Normal	41-60
TCGA-ED-A5KG-01	Normal	41-60
TCGA-ED-A66Y-01	Normal	41-60
TCGA-ED-A7PX-01	Normal	41-60
TCGA-ED-A7PY-01	Normal	20-40
TCGA-ED-A7PZ-01	Normal	61-80
TCGA-ED-A7XO-01	Normal	20-40
TCGA-ED-A7XP-01	Normal	41-60
TCGA-G3-A25U-01	Normal	61-80
TCGA-G3-A25Y-01	Normal	41-60
TCGA-G3-A25Z-01	Normal	41-60
TCGA-G3-A3CH-01	Normal	41-60
TCGA-G3-A3CK-01	Normal	61-80
TCGA-G3-AAV0-01	Normal	41-60
TCGA-G3-AAV1-01	Normal	41-60
TCGA-G3-AAV4-01	Normal	>80
TCGA-G3-AAV6-01	Normal	41-60
TCGA-G3-AAV7-01	Normal	20-40
TCGA-K7-A6G5-01	Normal	61-80
TCGA-O8-A75V-01	Normal	41-60
TCGA-QA-A7B7-01	Normal	41-60
TCGA-RC-A7S9-01	Normal	41-60
TCGA-RC-A7SB-01	Normal	41-60
TCGA-RC-A7SH-01	Normal	41-60
TCGA-UB-A7MC-01	Normal	41-60
TCGA-UB-A7ME-01	Normal	41-60
TCGA-UB-A7MF-01	Normal	41-60
TCGA-XR-A8TF-01	Deficiency	61-80
TCGA-ZP-A9CZ-01	Normal	61-80

Stage	Gender	Source
IIII	NA	AMC
III	NA	AMC
III	NA	AMC
IIII	NA	AMC
III	NA	AMC
IIII	NA	AMC
III	NA	AMC
III	NA	AMC
III	NA	AMC
III	NA	AMC
III	NA	AMC
III	NA	AMC
II	NA	AMC
III	NA	AMC
III	NA	AMC
II	NA	AMC
IIII	NA	AMC
III	NA	AMC
III	NA	AMC
II	NA	AMC
II	NA	AMC
III	NA	AMC
III	NA	AMC
III	NA	AMC
III	NA	AMC
III	NA	AMC
III	NA	AMC
II	NA	AMC
IIII	NA	AMC
III	NA	AMC
II	NA	AMC
III	NA	AMC
II	NA	AMC
II	NA	AMC
IIII	NA	AMC
IIII	NA	AMC
III	NA	AMC
III	NA	AMC
IIII	NA	AMC
III	NA	AMC
II	NA	AMC
III	NA	AMC
III	NA	AMC
III	NA	AMC
II	NA	AMC
IIII	NA	AMC
III	NA	AMC
III	NA	AMC
III	NA	AMC
IIII	NA	AMC
II	NA	AMC
IIII	NA	AMC
II	NA	AMC
II	NA	AMC
II	NA	AMC
III	NA	AMC
III	NA	AMC
III	NA	AMC
II	NA	AMC
II	NA	AMC
III	NA	AMC
III	NA	AMC

III	NA	AMC
III	NA	AMC
III	NA	AMC
II	NA	AMC
II	NA	AMC
III	NA	AMC
III	NA	AMC
II	NA	AMC
III	NA	AMC
III	NA	AMC
II	NA	AMC
II	NA	AMC
III	NA	AMC
IV	NA	AMC
III	NA	AMC
III	NA	AMC
III	NA	AMC
III	NA	AMC
II	NA	AMC
II	NA	AMC
II	NA	AMC
II	NA	AMC
III	NA	AMC
II	NA	AMC
II	NA	AMC
III	NA	AMC
II	NA	AMC
IV	NA	AMC
II	NA	AMC
II	NA	AMC
III	NA	AMC
III	NA	AMC
III	NA	AMC
III	NA	AMC
II	NA	AMC
II	NA	AMC
IV	NA	AMC
III	NA	AMC
III	NA	AMC
II	NA	AMC
IV	NA	AMC
III	NA	AMC
II	NA	AMC
III	NA	AMC
III	NA	AMC
III	NA	AMC
II	NA	AMC
II	NA	AMC
II	NA	AMC
III	NA	AMC
III	NA	AMC
III	NA	AMC
III	NA	AMC
III	NA	AMC
II	NA	AMC
III	NA	AMC
II	NA	AMC
II	NA	AMC
IV	NA	AMC
III	NA	AMC
II	NA	AMC
IV	NA	AMC
IV	NA	AMC
III	NA	AMC

II	NA	AMC
III	NA	AMC
III	NA	AMC
II	NA	AMC
II	NA	AMC
IV	NA	AMC
III	NA	AMC
III	NA	AMC
II	NA	AMC
III	NA	AMC
IV	NA	AMC
III	NA	AMC
III	NA	AMC
II	NA	AMC
III	NA	AMC
III	NA	AMC
IV	NA	AMC
III	NA	AMC
III	NA	AMC
II	NA	AMC
III	NA	AMC
III	NA	AMC
II	NA	AMC
II	NA	AMC
III	NA	AMC
III	NA	AMC
IV	NA	AMC
II	NA	AMC
IV	NA	AMC
II	NA	AMC
III	NA	AMC
II	NA	AMC
III	NA	AMC
III	NA	AMC
II	NA	AMC
IV	NA	AMC
III	NA	AMC
II	NA	AMC
III	NA	AMC
III	NA	AMC
II	NA	AMC
I	Male	CHCC
I	Male	CHCC
I	Female	CHCC
I	Female	CHCC
III	Female	CHCC
I	Male	CHCC
III	Male	CHCC
I	Female	CHCC
II	Male	CHCC
I	Male	CHCC
III	Male	CHCC
I	Male	CHCC
I	Male	CHCC
I	Male	CHCC
I	Male	CHCC
I	Male	CHCC
I	Male	CHCC
I	Female	CHCC
I	Male	CHCC
I	Male	CHCC
II	Male	CHCC
I	Male	CHCC
I	Male	CHCC

III	Male	CHCC
II	Male	CHCC
II	Male	CHCC
I	Male	CHCC
I	Male	CHCC
IV	Male	CHCC
II	Male	CHCC
III	Male	CHCC
III	Male	CHCC
III	Male	CHCC
I	Female	CHCC
III	Female	CHCC
III	Male	CHCC
I	Male	CHCC
III	Male	CHCC
I	Male	CHCC
I	Male	CHCC
I	Male	CHCC
IV	Male	CHCC
III	Male	CHCC
III	Male	CHCC
III	Male	CHCC
I	Female	CHCC
I	Male	CHCC
I	Male	CHCC
III	Male	CHCC
I	Male	CHCC
I	Male	CHCC
III	Male	CHCC
II	Male	CHCC
I	Male	CHCC
I	Male	CHCC
I	Female	CHCC
II	Male	CHCC
III	Male	CHCC
I	Female	CHCC
I	Male	CHCC
III	Male	CHCC
I	Male	CHCC
I	Male	CHCC
I	Female	CHCC
I	Male	CHCC
III	Male	CHCC
I	Male	CHCC
II	Male	CHCC
II	Male	CHCC
I	Male	CHCC
I	Female	CHCC
I	Male	CHCC
I	Male	CHCC
III	Female	CHCC
III	Male	CHCC
I	Male	CHCC
III	Male	CHCC
I	Male	CHCC
I	Male	CHCC
I	Male	CHCC
III	Male	CHCC
III	Male	CHCC
III	Male	CHCC
I	Male	CHCC
III	Male	CHCC
I	Male	CHCC
I	Female	CHCC

III	Male	CHCC
I	Male	CHCC
III	Male	CHCC
I	Male	CHCC
I	Male	CHCC
I	Female	CHCC
I	Male	CHCC
I	Male	CHCC
I	Male	CHCC
III	Female	CHCC
I	Male	CHCC
III	Male	CHCC
III	Male	CHCC
I	Male	CHCC
I	Male	CHCC
I	Male	CHCC
III	Male	CHCC
I	Female	CHCC
III	Male	CHCC
I	Female	CHCC
I	Female	CHCC
III	Female	CHCC
I	Female	CHCC
II	Male	CHCC
III	Female	CHCC
II	Female	CHCC
I	Male	CHCC
I	Female	CHCC
I	Male	CHCC
III	Male	CHCC
I	Female	CHCC
I	Female	CHCC
I	Male	CHCC
I	Male	CHCC
I	Male	CHCC
I	Male	CHCC
I	Male	CHCC
III	Male	CHCC
I	Male	CHCC
I	Male	CHCC
III	Male	CHCC
III	Male	CHCC
III	Male	CHCC
I	Male	CHCC
III	Male	CHCC
III	Male	CHCC
III	Male	CHCC
III	Male	CHCC
I	Female	CHCC
I	Male	CHCC
III	Male	CHCC
III	Male	CHCC
I	Female	CHCC
I	Male	CHCC
II	Female	CHCC
III	Male	CHCC
III	Male	CHCC
I	Female	CHCC
I	Female	CHCC
III	Male	CHCC
III	Male	CHCC
III	Male	CHCC
III	Male	CHCC
NA	Male	TCGA

IV	Female	TCGA
II	Male	TCGA
III	Female	TCGA
III	Male	TCGA
III	Male	TCGA
III	Male	TCGA
III	Male	TCGA
III	Male	TCGA
III	Male	TCGA
III	Male	TCGA
III	Male	TCGA
III	Male	TCGA
III	Male	TCGA
II	Male	TCGA
III	Male	TCGA
III	Male	TCGA
III	Male	TCGA
III	Female	TCGA
III	Male	TCGA
IV	Male	TCGA
I	Male	TCGA
III	Male	TCGA
I	Male	TCGA
II	Male	TCGA
I	Male	TCGA
III	Female	TCGA
II	Male	TCGA
I	Male	TCGA
I	Male	TCGA
I	Male	TCGA
I	Female	TCGA
I	Male	TCGA
I	Male	TCGA
I	Male	TCGA
II	Male	TCGA
II	Male	TCGA
I	Male	TCGA
II	Male	TCGA
I	Male	TCGA
I	Male	TCGA
II	Male	TCGA
I	Male	TCGA
I	Female	TCGA
I	Male	TCGA
I	Male	TCGA
I	Female	TCGA
I	Male	TCGA
III	Male	TCGA
I	Female	TCGA
I	Male	TCGA
I	Male	TCGA
I	Male	TCGA
I	Male	TCGA
I	Female	TCGA
I	Female	TCGA
II	Female	TCGA
I	Male	TCGA
I	Male	TCGA
I	Female	TCGA
III	Female	TCGA
I	Male	TCGA
I	Male	TCGA
I	Female	TCGA
I	Male	TCGA

I	Male	TCGA
I	Male	TCGA
II	Male	TCGA
I	Male	TCGA
I	Male	TCGA
I	Male	TCGA
I	Male	TCGA
II	Male	TCGA
II	Male	TCGA
I	Male	TCGA
II	Male	TCGA
I	Male	TCGA
I	Male	TCGA
II	Male	TCGA
II	Male	TCGA
II	Female	TCGA
III	Female	TCGA
II	Female	TCGA
II	Female	TCGA
II	Male	TCGA
III	Male	TCGA
II	Female	TCGA
I	Female	TCGA
I	Female	TCGA
I	Male	TCGA
III	Male	TCGA
I	Male	TCGA
I	Male	TCGA
III	Male	TCGA
I	Female	TCGA
III	Female	TCGA
II	Male	TCGA
I	Male	TCGA
I	Male	TCGA
II	Male	TCGA
I	Female	TCGA
II	Male	TCGA
II	Male	TCGA
III	Male	TCGA
I	Male	TCGA
III	Male	TCGA
I	Male	TCGA
NA	Male	TCGA
