# Supplementary material for: ARID1A deficiency promotes progression and potentiates therapeutic antitumour immunity in hepatitis B virus-related hepatocellular carcinoma
Source: BMC Gastroenterol. 2024 Jan 2;24:11. doi: 10.1186/s12876-023-03059-w (PMC10759659; doi:10.1186/s12876-023-03059-w)
Supplement: Supplementary file 2 — Additional file 2: Supplementary File S2. ssGSEA score of immune signatures. [file 12876_2023_3059_MOESM2_ESM.pdf]

Supplementary File S2. ssGSEA score of immune signatures

ID
T112
T113
T127
T141
T187
T191
T195
T211
T217
T221
T223
T227
T231
T257
T261
T267
T271
T277
T283
T285
T311
T313
T327
T331
T341
T343
T351
T353
T355
T357
T361
T363
T365
T367
T375
T383
T385
T387
T391
T393
T395
T411
T413
T415
T421
T423
T425
T427
T431
T433
T435
T443

T445
T451
T455
T461
T463
T465
T467
T471
T473
T477
T481
T483
T487
T491
T493
T497
T513
T515
T517
T523
T525
T527
T533
T537
T545
T553
T557
T563
T567
T571
T573
T615
T617
T627
T635
T641
T647
T661
T663
T665
T671
T685
T695
T713
T715
T721
T724
T727
T737
T741
T743
T745
T755
T777

T785
T813
T815
T817
T823
T851
T857
T861
T863
T865
T867
T873
T877
T881
T883
T911
T913
T915
T917
T921
T923
T925
T937
T943
T951
T953
T955
T957
T963
T965
T967
T975
T977
T981
T983
T1013
T1015
T1021
T1025
T1027
T1031
T1041
T1043
T1045

aDC	all_immune	B
0.332933771	0.56546765	0.39104621
0.589334096	0.71086738	0.43390667
0.337228129	0.6145476	0.45864836
0.400491022	0.73501396	0.46830115
0.368848393	0.73009115	0.5274511
0.415569278	0.74697821	0.55132946
0.410947778	0.72651244	0.53127348
0.558208627	0.87185564	0.60899204
0.553823635	0.81497119	0.57576365
0.396000824	0.66772396	0.46018835
0.436365382	0.7747629	0.57052757
0.3195842	0.66205514	0.45908054
0.352919006	0.646624	0.41246147
0.323987886	0.57644307	0.38016646
0.413042305	0.6921198	0.43901172
0.343433062	0.60460248	0.37611289
0.261646875	0.60127791	0.39564564
0.384239522	0.63402362	0.47444315
0.457279852	0.70267161	0.37889954
0.367393121	0.68429214	0.46250064
0.426277314	0.82406183	0.53015992
0.322817755	0.57894515	0.36566238
0.44630505	0.67711989	0.45478724
0.274400766	0.56246337	0.40682405
0.384902185	0.63140603	0.48551951
0.574429855	0.65004847	0.4668582
0.3630818	0.5497472	0.36823152
0.388544855	0.67902936	0.46724424
0.364328675	0.58606647	0.41925726
0.332193466	0.53344013	0.39773078
0.287886821	0.6578945	0.40758228
0.293907509	0.70472485	0.45799116
0.376173915	0.73256205	0.48910772
0.389068095	0.680314	0.46265435
0.412018556	0.68203975	0.47661719
0.41080197	0.77521293	0.54388916
0.398982977	0.74469757	0.52017394
0.370480519	0.68098325	0.48686736
0.394933536	0.70905457	0.42934987
0.358395431	0.5674999	0.38904906
0.388494414	0.68502823	0.49426568
0.368800748	0.56194335	0.39152425
0.381047943	0.66090174	0.44945745
0.493444107	0.77141862	0.54266128
0.403428272	0.68072204	0.4831629
0.459790062	0.66620536	0.49764528
0.255912001	0.57825198	0.43062775
0.350799386	0.63867287	0.44430434
0.358445563	0.61204081	0.42844751
0.335007373	0.65439235	0.4846221
0.227416287	0.53172041	0.34403226
0.340295688	0.65002527	0.429106

0.383848585	0.56282324	0.37483593
0.50850382	0.78060778	0.52057695
0.420740624	0.73054314	0.51345609
0.410741296	0.69665809	0.4951732
0.259351079	0.60706289	0.45898251
0.32226794	0.57873394	0.37585509
0.524386456	0.73323981	0.49975878
0.491727128	0.75018632	0.51528965
0.231934065	0.53478355	0.33850097
0.301448391	0.58898955	0.3559103
0.283569155	0.68504995	0.4794718
0.367103769	0.66968177	0.47274014
0.381625727	0.65326186	0.42184943
0.347562395	0.71989996	0.45168806
0.42285193	0.68856652	0.52709
0.374168671	0.60371271	0.43663531
0.322874597	0.62754314	0.3996156
0.300881936	0.61705626	0.39719544
0.327493275	0.60303295	0.36557469
0.27258459	0.52192565	0.36982703
0.3878807	0.70582554	0.46912807
0.442282	0.63104202	0.41612037
0.343826438	0.60150999	0.39220659
0.350903387	0.63483974	0.43559478
0.299343405	0.64345997	0.46644133
0.42891875	0.70738198	0.43918088
0.528534712	0.70913633	0.43624705
0.399838867	0.75803299	0.54399925
0.383270855	0.63784143	0.36608579
0.342425705	0.63548322	0.44274457
0.449032603	0.76095297	0.51713355
0.264898456	0.57820586	0.39437973
0.313968313	0.68456858	0.51851292
0.414428181	0.64619209	0.40279743
0.309535491	0.5906283	0.42567065
0.271887687	0.68696051	0.48078034
0.315066222	0.57754358	0.36521452
0.473705388	0.74796514	0.56680409
0.277622347	0.62881039	0.39927361
0.369429687	0.6272864	0.37626636
0.336572712	0.55481441	0.44158124
0.446137163	0.68502541	0.4698802
0.250402378	0.57509157	0.36530051
0.361276732	0.72270783	0.48915275
0.31766885	0.672756408	0.345534343
0.50796879	0.80025437	0.58042457
0.460645193	0.81709446	0.61389837
0.539879649	0.64227828	0.46155607
0.358187943	0.59809153	0.43307782
0.322038662	0.58719951	0.40832287
0.13022423	0.44612407	0.3312553
0.335460675	0.60672774	0.41331873
0.398440963	0.6556619	0.49483903
0.241375148	0.55323092	0.42475002

0.272741947	0.62336993	0.45618174
0.191722563	0.58390959	0.43117844
0.385155843	0.62392259	0.528251
0.401941132	0.66447411	0.47747658
0.367868366	0.73058917	0.54705318
0.462783524	0.71578437	0.44634472
0.340500116	0.71806654	0.51615996
0.436674693	0.76760706	0.48506409
0.323454064	0.64746928	0.47772471
0.378295315	0.58896154	0.44015605
0.353643826	0.7271425	0.50818427
0.298502535	0.59554478	0.43587503
0.24593842	0.56601551	0.40577099
0.302490105	0.66010418	0.48095794
0.395302252	0.65407862	0.4774668
0.142481665	0.55683005	0.35321321
0.270208976	0.6574964	0.43184209
0.377265556	0.68155174	0.45770248
0.265656011	0.63298412	0.43430727
0.308411573	0.56778269	0.41920639
0.369818894	0.7045259	0.45478845
0.326678534	0.67540464	0.48189404
0.361930061	0.67740284	0.45925414
0.281824223	0.67483946	0.50546146
0.400719126	0.58065015	0.41566561
0.418608712	0.59752354	0.40913068
0.429339196	0.68833287	0.5038345
0.349908781	0.57406075	0.35439971
0.325718771	0.68021717	0.49981589
0.178175673	0.51812617	0.34947674
0.30873142	0.6219491	0.41673291
0.328438146	0.6068692	0.44247417
0.376095443	0.57222005	0.41221007
0.265471135	0.55965191	0.30260836
0.361457927	0.66193295	0.40550351
0.334996449	0.53653249	0.30196361
0.42581984	0.52362252	0.39793513
0.325845347	0.54974884	0.38647703
0.261954001	0.56357836	0.39023513
0.300285263	0.53141834	0.34714384
0.457423555	0.74830187	0.53773969
0.302208532	0.68904749	0.46896983
0.484759986	0.76494834	0.53819364
0.290298961	0.62476962	0.43809934

CD8T	Cytotoxic	DC	Eosinophils	iDC	Macrophages	Mast
0.70427598	0.5068665	0.41846236	0.54725685	0.50457919	0.52168333	0.3194326
0.73123388	0.66462778	0.53036533	0.52488574	0.53984281	0.57291333	0.32523286
0.70424512	0.49936635	0.44094454	0.51327414	0.53048052	0.58303668	0.16488269
0.69595295	0.59808863	0.652461	0.49288972	0.56439312	0.70662504	0.30228157
0.71797743	0.62451636	0.52755947	0.50298623	0.57800337	0.65225232	0.32778641
0.71518962	0.60509826	0.55506741	0.47986032	0.55762296	0.71625536	0.31404239
0.71722095	0.59183905	0.55894865	0.53752475	0.58056396	0.67536333	0.32293141
0.77276818	0.70015613	0.62226027	0.48337586	0.59251066	0.71227493	0.33467447
0.72455404	0.6605207	0.54891071	0.55166901	0.56967147	0.67255723	0.25303606
0.72709881	0.5258738	0.4111131	0.52787317	0.5657458	0.6345632	0.26434703
0.7376845	0.6560444	0.51145074	0.52875252	0.65833709	0.63965195	0.39219964
0.70086588	0.55656802	0.40984114	0.52570005	0.54862587	0.59868659	0.55476612
0.69040944	0.45929733	0.49300717	0.58417443	0.55958698	0.5880478	0.31341719
0.67548862	0.45512689	0.43482233	0.48195914	0.47518152	0.61957089	0.33418478
0.70597343	0.60599089	0.46075218	0.56016775	0.55930906	0.56415356	0.38663329
0.68843763	0.55002707	0.41501448	0.51456551	0.47341515	0.57983098	0.30329615
0.71541321	0.53498611	0.36284526	0.54256626	0.45522222	0.51751573	0.27500259
0.7027129	0.52254176	0.40708495	0.54055377	0.51579552	0.55171237	0.42235774
0.67222357	0.50150836	0.35299062	0.51835962	0.57516783	0.74597229	0.34932922
0.6893914	0.513058	0.42656286	0.49984852	0.58946642	0.65069299	0.28965608
0.73124495	0.60552336	0.6680485	0.54744568	0.65752836	0.78217425	0.50405721
0.66890297	0.45178298	0.46517104	0.48962223	0.48520968	0.61049841	0.31163193
0.70060349	0.57216774	0.54314962	0.55839762	0.58300755	0.58408109	0.36355304
0.71568293	0.44850675	0.3146977	0.54660223	0.52942646	0.57263596	0.25311017
0.70036449	0.53776598	0.51980955	0.55232394	0.57703671	0.57294585	0.26099906
0.7019591	0.49725622	0.53690621	0.54747227	0.52988175	0.66245483	0.30380709
0.69459164	0.39046207	0.34588736	0.49834848	0.49449101	0.57780649	0.2211868
0.72994815	0.62316146	0.52932293	0.54710468	0.56370008	0.58313324	0.34124385
0.68652211	0.41931413	0.47369073	0.5025829	0.49232649	0.56335806	0.32956604
0.67840298	0.47486026	0.41891118	0.56782565	0.45388618	0.53192314	0.29917608
0.70647635	0.51436579	0.45287738	0.53630761	0.5648332	0.61994487	0.34027317
0.72558675	0.58186777	0.44194571	0.53207503	0.59484447	0.69687353	0.49405673
0.69120922	0.58167423	0.58676303	0.51963304	0.60263173	0.68831435	0.31046284
0.7101755	0.54890259	0.42725855	0.51037108	0.55454957	0.62396577	0.42974394
0.65364084	0.48791385	0.41854171	0.49216634	0.48986615	0.63179941	0.33610909
0.75327171	0.72845098	0.5453421	0.50713532	0.56757911	0.66908794	0.32666713
0.76851428	0.65515993	0.48257034	0.50722249	0.5485567	0.65868936	0.29383802
0.67948397	0.54142808	0.40905016	0.50301731	0.5524312	0.61608244	0.29820301
0.69102766	0.49797081	0.51042631	0.51290241	0.58976165	0.64485236	0.28559519
0.69686366	0.51876247	0.4446532	0.54291	0.50235969	0.52700375	0.26299454
0.70682827	0.61609399	0.53748971	0.53177967	0.58943467	0.55720738	0.33195891
0.67914123	0.50371606	0.47991357	0.56051433	0.48820482	0.51480333	0.35541749
0.72072295	0.5395249	0.43947179	0.52144021	0.56191357	0.61388321	0.27124203
0.75483179	0.66557118	0.54612485	0.50831152	0.61473438	0.68963914	0.38099335
0.70899853	0.55642662	0.54154107	0.542672	0.57309833	0.70831166	0.40596991
0.70697044	0.64003722	0.474331	0.53110844	0.57618801	0.59530475	0.41702287
0.67976982	0.51763508	0.27755593	0.52179049	0.51628367	0.55527428	0.23760904
0.71330455	0.52793076	0.45331488	0.50342611	0.5345494	0.62818234	0.37972231
0.71568511	0.57488345	0.45817153	0.53558232	0.5199051	0.56528368	0.28686508
0.70210317	0.50158793	0.45659836	0.53429503	0.54740475	0.61743982	0.3381778
0.66692754	0.39706132	0.41253709	0.56367716	0.48244645	0.49577171	0.29775533
0.71980332	0.58344572	0.42138867	0.51440032	0.51523022	0.59283393	0.28490514

0.65499752	0.45055082	0.49395852	0.57045458	0.48956781	0.53832283	0.24592822
0.76862797	0.71347043	0.54465494	0.54727259	0.54382621	0.61697582	0.23262583
0.70747179	0.53094058	0.5356835	0.52296925	0.59439229	0.65019082	0.23498995
0.72591506	0.58239336	0.55536544	0.51401189	0.58571698	0.63644313	0.30283336
0.67071391	0.46740942	0.36072005	0.52597904	0.50019998	0.6052466	0.32975691
0.66855565	0.44750714	0.48268798	0.5320616	0.53456855	0.52419654	0.30772342
0.7349471	0.61100705	0.44959296	0.51160984	0.58046161	0.61893288	0.29002807
0.75284443	0.63858759	0.54022893	0.51149646	0.56740411	0.61283884	0.28610252
0.65016943	0.38806018	0.34262333	0.53581854	0.4371395	0.49477657	0.24992251
0.70255933	0.48064746	0.40979174	0.52596882	0.50153795	0.52939386	0.27168272
0.73415479	0.68666009	0.52697544	0.53938144	0.54422788	0.57553823	0.31072592
0.71025621	0.53305526	0.58781592	0.50572688	0.59684547	0.62348231	0.35248614
0.67582478	0.54086297	0.52210979	0.54363683	0.51404337	0.59289517	0.24058826
0.70971684	0.52551878	0.55541453	0.52688473	0.5733327	0.69481971	0.30527085
0.710844	0.55711052	0.51134191	0.53029058	0.56138995	0.60512291	0.32350918
0.69802715	0.48277307	0.350948	0.53610953	0.4991347	0.54967102	0.30268524
0.68687267	0.61965482	0.5265813	0.57371985	0.51663838	0.5813352	0.35936056
0.70228187	0.55557803	0.5351705	0.52364149	0.52374234	0.59531762	0.29281938
0.6826528	0.4140357	0.46443499	0.516515	0.52827868	0.59322388	0.31777685
0.66990615	0.51651669	0.35676445	0.54153199	0.41795863	0.45136722	0.27653645
0.72084526	0.5286453	0.48852374	0.53356491	0.5926842	0.65368701	0.33650511
0.69232994	0.48948391	0.51186725	0.55186614	0.52833247	0.58900907	0.37307296
0.66460775	0.47651733	0.44096839	0.52686562	0.52541402	0.59033704	0.29495947
0.68855028	0.46061393	0.4566434	0.51791239	0.53044605	0.64640281	0.29497432
0.71603782	0.49869882	0.47237067	0.53138572	0.55087938	0.62070253	0.33537567
0.7303172	0.56223721	0.51270638	0.53441539	0.58795997	0.61569455	0.28603125
0.72050909	0.55619168	0.5728494	0.53373465	0.58506612	0.6058168	0.30067946
0.71291157	0.61652546	0.57692152	0.53472958	0.65635943	0.6857494	0.37567859
0.66427647	0.39960536	0.41769074	0.5397068	0.55167959	0.64350117	0.33804126
0.7102668	0.47843602	0.38046479	0.53011439	0.54530039	0.6031569	0.51701138
0.71977143	0.57736914	0.53087684	0.50595943	0.57873362	0.71334758	0.40379892
0.67196726	0.51373488	0.4414497	0.51268966	0.50115366	0.49922275	0.26824343
0.72413213	0.59531326	0.53433498	0.51877735	0.59251483	0.57933792	0.21258252
0.69726572	0.6070367	0.4729765	0.55016665	0.53583618	0.55769576	0.30343001
0.67476134	0.50650923	0.47510365	0.52695397	0.51965493	0.51218084	0.24955265
0.73653028	0.61242594	0.36707131	0.52997361	0.54823482	0.56939384	0.25924562
0.66310979	0.47497268	0.40334748	0.52562461	0.50001765	0.51797098	0.27455047
0.69081038	0.63212221	0.44285971	0.50567781	0.54685842	0.61770688	0.26557518
0.68650966	0.52235569	0.31984586	0.47523716	0.41962523	0.49930432	0.17591979
0.71028651	0.46121172	0.55865482	0.52758181	0.5527151	0.60275504	0.33860207
0.72383785	0.38619048	0.46434532	0.56843566	0.51076614	0.55766221	0.35126769
0.72019226	0.54590493	0.56159445	0.57826066	0.55307846	0.56609228	0.29082953
0.67428614	0.40753497	0.39728385	0.50158436	0.52052224	0.50610633	0.25644651
0.7207794	0.59648903	0.51061648	0.54904953	0.58190978	0.67037913	0.52942991
0.725905893	0.566635139	0.438831304	0.539219335	0.564130276	0.621594972	0.337311836
0.77240844	0.77219303	0.52622104	0.53268333	0.6076919	0.5748794	0.3040599
0.74708067	0.70880382	0.48853719	0.48213191	0.53437909	0.63953995	0.20759657
0.7025239	0.61167525	0.4766369	0.5753406	0.53790689	0.56122909	0.2817953
0.67949642	0.48753251	0.46697645	0.58403192	0.53886125	0.59998689	0.32568118
0.6992474	0.47433774	0.43679219	0.54043124	0.53443637	0.54814205	0.28513221
0.63983947	0.28238526	0.27210905	0.53223347	0.46597498	0.51362621	0.20607276
0.67616748	0.45309374	0.43586668	0.54113166	0.51562003	0.57254264	0.28845784
0.70048476	0.58773497	0.47404824	0.53965718	0.54502243	0.54350732	0.28094993
0.65654465	0.46641101	0.4892494	0.53954138	0.49690604	0.54975329	0.30850054

0.6984619	0.56013532	0.4681471	0.49963615	0.52039221	0.53681417	0.25359771
0.68153214	0.3820428	0.39225334	0.53562513	0.49009117	0.55291275	0.24540791
0.64848673	0.46938662	0.46391475	0.52267707	0.53368672	0.56384293	0.24525028
0.71813191	0.48297791	0.4414609	0.5426078	0.55954586	0.61834831	0.28251863
0.71215283	0.60384277	0.49220066	0.52583933	0.55420643	0.62439213	0.32503359
0.68848522	0.52681749	0.53580619	0.54883888	0.56818712	0.64791973	0.26826264
0.73289725	0.62446689	0.48974702	0.53086666	0.54548673	0.58456218	0.30304123
0.7586743	0.67423635	0.44423757	0.55678589	0.5380554	0.58166864	0.25918744
0.67662061	0.51130606	0.49691097	0.54936961	0.53761816	0.61267284	0.30709726
0.67553255	0.50997075	0.38864943	0.50598433	0.47375543	0.46749523	0.20491665
0.73326697	0.6557202	0.4684457	0.57664877	0.54350455	0.6106093	0.28046583
0.68162303	0.49545802	0.43498321	0.49396351	0.53667666	0.51724933	0.30760909
0.71610845	0.4072974	0.17760466	0.50518173	0.49572573	0.49151931	0.37642276
0.71716395	0.53640983	0.46570024	0.53095142	0.52755243	0.52126325	0.25089629
0.67476955	0.48861156	0.44944346	0.50035728	0.52122693	0.58699401	0.19991022
0.69043593	0.50164374	0.39633485	0.51811303	0.46507769	0.49780691	0.27922005
0.71820984	0.60249282	0.48674379	0.50702009	0.5003331	0.53287688	0.21135279
0.72492315	0.62162581	0.44880218	0.56148149	0.52375892	0.51722832	0.27897637
0.73028776	0.48076743	0.32945108	0.48705091	0.48186962	0.52118159	0.39242427
0.65112018	0.40780715	0.3311815	0.50971946	0.51045935	0.54623089	0.26876191
0.70804258	0.54434847	0.51995395	0.55083717	0.57990777	0.64779503	0.31800547
0.69232885	0.56640318	0.47463533	0.46653126	0.54792772	0.54842366	0.24136455
0.65744985	0.51328961	0.46523254	0.51174973	0.55926191	0.55761314	0.30393328
0.69131975	0.47483816	0.39068806	0.50313333	0.53478656	0.60826504	0.30306846
0.67938407	0.44191517	0.34225936	0.51197416	0.48812094	0.51290529	0.21052925
0.6665443	0.47994643	0.43178219	0.52042073	0.48547672	0.53619141	0.28961615
0.73781358	0.6703847	0.4829969	0.56480336	0.52789383	0.55637399	0.26674071
0.67439498	0.4020351	0.38807282	0.52114601	0.46344483	0.53439143	0.29837346
0.70271086	0.55614572	0.4730342	0.51117334	0.5144183	0.54668323	0.22700406
0.64953507	0.31637551	0.27678932	0.56304693	0.47726816	0.49372276	0.25132252
0.70316176	0.53705325	0.34836739	0.50823142	0.49800498	0.54220402	0.3017523
0.69337198	0.52618708	0.43791909	0.5397779	0.5201009	0.52007991	0.25588196
0.69330022	0.46867285	0.37019506	0.54827305	0.46927814	0.47545024	0.20897287
0.69230403	0.48689681	0.44097226	0.55168117	0.4982986	0.47315821	0.22327941
0.65904625	0.37354374	0.37495735	0.5065351	0.56371585	0.56667953	0.27071356
0.62539777	0.33819665	0.42322372	0.49481328	0.48845844	0.49748323	0.20926272
0.64833857	0.32461911	0.36261859	0.49669955	0.47480048	0.46314667	0.18626588
0.67323862	0.38389168	0.42719963	0.54471772	0.49675564	0.51781807	0.26410456
0.67236417	0.43506664	0.36194562	0.53295239	0.5046526	0.5233363	0.25787941
0.68721489	0.46261898	0.4990213	0.53646996	0.49762562	0.56124484	0.29739702
0.73817252	0.69991008	0.37955701	0.52372264	0.49221607	0.55393994	0.18657108
0.73155734	0.57355927	0.38308859	0.53669059	0.5467854	0.51800371	0.30275932
0.73824588	0.69006279	0.43963515	0.51562617	0.48482777	0.55558554	0.19886421
0.68170552	0.47844544	0.4465429	0.49474907	0.49823949	0.54569499	0.23797052

Neutrophils	NKCD56brigh	NKCD56dim	NK	pDC	T	Thelper
0.36355203	0.4842735	0.24121816	0.50789908	0.47358749	0.38694158	0.73653523
0.46186292	0.47776211	0.4135594	0.53917222	0.46158181	0.58766742	0.75097178
0.44375332	0.53527087	0.26320646	0.52375082	0.42115451	0.40538739	0.71516267
0.47074351	0.50959258	0.22326365	0.55224729	0.49152252	0.59111585	0.7648943
0.47789182	0.49664287	0.33705049	0.58646663	0.54934581	0.56891874	0.73606477
0.48652481	0.43863643	0.23416311	0.51763565	0.72296271	0.59262845	0.74280438
0.50222941	0.5135932	0.24064039	0.54508002	0.45187926	0.55922137	0.71800911
0.43057566	0.54311133	0.49164606	0.57874476	0.40811976	0.71999325	0.82456663
0.58125458	0.51104342	0.30680671	0.5353184	0.30996717	0.65423444	0.78225612
0.44263364	0.50410728	0.1928656	0.54638314	0.38744058	0.53278511	0.75952281
0.55126291	0.52086196	0.26969958	0.56485663	0.49083648	0.64528531	0.72520895
0.44247499	0.49818732	0.27461457	0.54076155	0.49078747	0.47167832	0.75623101
0.48320187	0.52368781	0.20135825	0.49899735	0.39915225	0.37449911	0.72549725
0.44551696	0.5093798	0.18944525	0.52333721	0.35416769	0.33943597	0.71658495
0.4989638	0.53129379	0.22921527	0.56124939	0.52896065	0.47496015	0.7238495
0.40244506	0.54697192	0.2039412	0.49893129	0.3683785	0.40538383	0.71984597
0.36677169	0.51247294	0.17376964	0.49813755	0.40165139	0.42634771	0.74779579
0.40788416	0.5144639	0.24023313	0.54889925	0.5052923	0.53215762	0.75580502
0.51617136	0.54729393	0.29967937	0.57694175	0.3000686	0.39078629	0.73404364
0.50722563	0.54358201	0.32036558	0.49100998	0.47476356	0.49940581	0.75313118
0.60031568	0.62232803	0.35185859	0.56749442	0.48275102	0.57574156	0.73953905
0.40385992	0.5853044	0.19082898	0.529034	0.41150096	0.36053925	0.73107108
0.54029761	0.52263569	0.30985938	0.53573672	0.51024158	0.49701474	0.72708982
0.3576203	0.49493361	0.10356401	0.49365965	0.40023031	0.39652868	0.73841076
0.3831796	0.43431184	0.2121373	0.5322746	0.34309306	0.48155987	0.73641801
0.4175316	0.59088635	0.26843811	0.50452121	0.39273289	0.41918266	0.75012057
0.39399646	0.5833536	0.20874968	0.51621904	0.2456755	0.35126251	0.72193284
0.49702257	0.48089929	0.34193669	0.55119887	0.46814818	0.5026567	0.74025469
0.34811867	0.45952439	0.16580945	0.52566757	0.38655853	0.37040647	0.72770945
0.3843723	0.52857773	0.20249285	0.51005831	0.41728329	0.35140773	0.70748244
0.46353026	0.53126855	0.14566245	0.55866796	0.45545646	0.46788646	0.73057646
0.43140556	0.54249651	0.23260766	0.52442799	0.51568089	0.50200188	0.75403127
0.48595137	0.53119339	0.24844081	0.55172117	0.50646837	0.54286031	0.73972206
0.38976661	0.5452834	0.28910433	0.50065857	0.40478757	0.52276689	0.78391371
0.42239646	0.44932596	0.26447583	0.52344749	0.31741559	0.43224756	0.71768478
0.45995838	0.47958883	0.25933888	0.53952077	0.48343706	0.60670559	0.76497998
0.44227899	0.59751007	0.37298535	0.5529381	0.48985642	0.63520395	0.79353427
0.40286474	0.51754381	0.31658826	0.48847866	0.35029647	0.53254588	0.75901298
0.44331614	0.53952254	0.25837032	0.51487444	0.34799334	0.53123708	0.78016597
0.43749893	0.48047238	0.12082001	0.55148563	0.4139511	0.33894607	0.69077214
0.50502175	0.56093013	0.38388175	0.52126556	0.51278973	0.50987193	0.68590878
0.43069132	0.5379957	0.21623558	0.52187476	0.48186897	0.39362748	0.71555705
0.42287147	0.51808577	0.24641829	0.52594598	0.45888666	0.51881422	0.74504418
0.48529414	0.50177432	0.3075941	0.58021478	0.39052776	0.62183485	0.74496293
0.44731532	0.5121086	0.21361339	0.54812714	0.57213211	0.52651706	0.75192104
0.45549129	0.50315504	0.27794451	0.57702921	0.36597736	0.46466817	0.70668295
0.4097986	0.55447715	0.22474478	0.50465228	0.3525996	0.41688702	0.7471948
0.45595437	0.58636352	0.18736689	0.53298171	0.39430098	0.41382978	0.75545217
0.36918189	0.45427656	0.25236413	0.55840625	0.49803989	0.43131031	0.73867287
0.45749581	0.55164914	0.16315762	0.56677498	0.31741559	0.49608218	0.73707812
0.37504173	0.43699469	0.30356566	0.5139302	0.4211055	0.29623389	0.71544911
0.39037322	0.50637919	0.37577682	0.52644061	0.47309747	0.49457055	0.7487994

0.3943584	0.5095189	0.19935917	0.52338822	0.40047533	0.3923964	0.72026024
0.50077988	0.54281943	0.32315402	0.55374034	0.657887	0.65387843	0.77806876
0.44054037	0.58207619	0.21848802	0.56731439	0.41649924	0.57090142	0.75581388
0.46026083	0.56210175	0.28209263	0.56026253	0.40718871	0.55404221	0.72517481
0.38352667	0.54464586	0.13278904	0.53524534	0.34108394	0.46434691	0.74545334
0.37985613	0.48871298	0.26975532	0.5210519	0.41032489	0.35790223	0.71919012
0.48922912	0.53070459	0.35109153	0.54366098	0.4314941	0.56992033	0.74773246
0.40585489	0.55173395	0.31776435	0.55997596	0.52655951	0.64629038	0.77240398
0.35816696	0.55451721	0.15764619	0.51238603	0.41272603	0.3289184	0.72364678
0.37093707	0.5076048	0.20771069	0.51961729	0.42566276	0.385609	0.72831206
0.52151874	0.46338734	0.30066748	0.55628814	0.40135738	0.45806034	0.6773621
0.45195669	0.49468947	0.23423533	0.55767454	0.47853678	0.49352272	0.69497131
0.40969069	0.46083432	0.27499238	0.52997345	0.50450826	0.51097722	0.7464583
0.50062575	0.55719822	0.26579273	0.55573549	0.66273828	0.47937259	0.73633543
0.45960009	0.49634772	0.298418	0.54241534	0.4262508	0.53638589	0.72551948
0.4551737	0.51196353	0.24418611	0.54002523	0.40968785	0.42340238	0.75182732
0.46820935	0.48261182	0.28130557	0.56626628	0.53199882	0.46725476	0.70441973
0.43391764	0.52126377	0.15378818	0.53819303	0.55978341	0.43467736	0.6998497
0.42075019	0.51668354	0.22881122	0.52590612	0.4526143	0.41349033	0.73814634
0.42520818	0.52248323	0.21565486	0.50858064	0.36695742	0.3185315	0.67727754
0.48300227	0.49279969	0.15248457	0.54071166	0.47334248	0.47150362	0.75236371
0.45800755	0.5151491	0.22456042	0.53881269	0.43693341	0.45684552	0.75245224
0.44926438	0.4979051	0.27410666	0.50933566	0.50416524	0.32757732	0.70320275
0.50169723	0.51131914	0.20584827	0.50453731	0.48333905	0.39662484	0.71741049
0.42827273	0.50855029	0.12859205	0.54373233	0.44193169	0.4453308	0.70141691
0.49711217	0.54460341	0.32512168	0.54873519	0.42928897	0.50842739	0.76054667
0.51991116	0.52972673	0.27677818	0.52193157	0.57257314	0.51580178	0.72222471
0.51610669	0.57783789	0.34306484	0.5645626	0.43933454	0.604239	0.72193216
0.537273	0.57108961	0.14742836	0.52653637	0.43521831	0.37141974	0.74114789
0.42959885	0.49373838	0.29001945	0.53044251	0.42022345	0.42197228	0.73623419
0.53793389	0.47432512	0.38219225	0.5336431	0.4192924	0.54578949	0.77388077
0.44917089	0.52304722	0.25485197	0.53971357	0.39523203	0.35477047	0.68233558
0.43036993	0.555818	0.30698405	0.53992133	0.42997501	0.53051523	0.69847136
0.48294828	0.54442404	0.31652641	0.55459592	0.50490028	0.48688908	0.70323285
0.41394461	0.58669647	0.23307749	0.52827688	0.49015044	0.42978021	0.6954441
0.38106437	0.61027512	0.34668725	0.53682356	0.40753173	0.51752221	0.72898824
0.34541819	0.4461261	0.13404738	0.53606043	0.35014946	0.38243894	0.71368314
0.4055456	0.62649538	0.42921925	0.54883496	0.37945313	0.58179451	0.76038323
0.3685793	0.49135476	0.0968919	0.5125596	0.34701328	0.44645065	0.72803938
0.48872716	0.45476578	0.13613948	0.53345087	0.37102465	0.41319801	0.76142811
0.41267068	0.66824398	0.18012735	0.52091949	0.33613466	0.38498849	0.70674404
0.49237761	0.4891201	0.21280259	0.55412754	0.39459499	0.53633165	0.73988251
0.37114014	0.45214649	0.09545381	0.54457002	0.36249816	0.36130764	0.72161743
0.46911921	0.54525071	0.18844067	0.54010093	0.5297447	0.5164561	0.73113245
0.479734885	0.584120913	0.384967098	0.559703566	0.353579654	0.394846588	0.685962812
0.45368497	0.64415128	0.37111693	0.59280394	0.40650267	0.65726148	0.71894946
0.43356041	0.56795557	0.34793519	0.52688662	0.44315676	0.72789546	0.79894469
0.38470642	0.57401779	0.38466792	0.52510899	0.37783604	0.49543224	0.7307172
0.4270588	0.53199589	0.18599182	0.552038	0.42066448	0.46553024	0.75003402
0.42881702	0.46244696	0.30554066	0.53644344	0.38895967	0.40207733	0.74990555
0.30268934	0.51985171	0.15420107	0.52292106	0.46888323	0.21728615	0.69874474
0.4734043	0.44236716	0.18450345	0.50634798	0.38807762	0.42743702	0.70113872
0.4029905	0.48063827	0.18359405	0.53829103	0.40841378	0.50748088	0.71040235
0.43433246	0.56694826	0.24362417	0.54251716	0.42992601	0.37563133	0.6818336

0.42675063	0.5546232	0.14059038	0.5455604	0.41713628	0.45846045	0.67179293
0.37825846	0.62951088	0.11654893	0.52064477	0.33956486	0.35481405	0.69892981
0.39344475	0.54102699	0.20519168	0.51948087	0.29565835	0.42710787	0.70765145
0.41558449	0.57331194	0.24071568	0.55684309	0.46898123	0.45695864	0.73852231
0.45476129	0.5626234	0.30163271	0.55908539	0.46060175	0.55114571	0.73155853
0.5026161	0.50633934	0.35179579	0.50048005	0.39699613	0.46045421	0.72002138
0.46094933	0.58598464	0.30734683	0.56520168	0.40669868	0.55413992	0.70788429
0.4599119	0.50872357	0.40215982	0.52226991	0.47780173	0.62825052	0.77136465
0.38734976	0.51078932	0.15168587	0.53380816	0.49260058	0.41260528	0.75338013
0.41037248	0.52507137	0.21806055	0.50163336	0.35598079	0.43598784	0.70605302
0.43400465	0.53780803	0.32671308	0.56471214	0.39939727	0.58581502	0.75109972
0.41644477	0.56479009	0.16544914	0.53436891	0.26478659	0.41781875	0.70153833
0.40827552	0.66556576	0.22759218	0.54213114	0.21705787	0.42863453	0.72554397
0.38710005	0.58302961	0.15095193	0.56926928	0.40282746	0.50766193	0.69528673
0.39778024	0.63215601	0.29205397	0.54223856	0.39268388	0.495481	0.75838741
0.39728383	0.49761229	0.27449712	0.49277874	0.26160141	0.3322033	0.70626556
0.41266536	0.54544528	0.31251719	0.52671066	0.29639339	0.47530615	0.68371721
0.45971058	0.54933073	0.33627383	0.53645053	0.3842554	0.50849791	0.73519724
0.42239116	0.65845076	0.27152429	0.55428584	0.36293919	0.46593328	0.75411535
0.36266029	0.53532127	0.22314057	0.53180158	0.39395796	0.35624114	0.71513055
0.47228466	0.62994419	0.30390398	0.57408253	0.4350713	0.49652595	0.73634419
0.36894693	0.57849159	0.28094538	0.51587989	0.3140344	0.51739369	0.70016371
0.44741787	0.47857547	0.28946743	0.53230086	0.35181555	0.44480905	0.69788248
0.49900629	0.57252739	0.28680897	0.55635558	0.3859215	0.45073471	0.72292491
0.31999222	0.55951523	0.15963659	0.48877283	0.30663498	0.39800079	0.72942881
0.43588212	0.50154027	0.2970863	0.51491827	0.3351546	0.3343477	0.72916459
0.46996283	0.52722235	0.31683894	0.5477881	0.11209389	0.5093164	0.71518977
0.40567416	0.47452545	0.25910503	0.48732553	0.41424511	0.27027606	0.69353091
0.3968402	0.56717069	0.22046319	0.53245864	0.3157495	0.53935928	0.73457197
0.35728383	0.58189595	0.1308652	0.51047159	0.38043318	0.29193462	0.73429442
0.36856207	0.5753708	0.2642669	0.51745381	0.35627481	0.42314543	0.73248056
0.31575428	0.57058279	0.27008722	0.52037339	0.42541775	0.42274826	0.72109524
0.40279445	0.486617	0.20218032	0.51163315	0.34500417	0.35458715	0.75592834
0.43913006	0.45645867	0.19483094	0.51813798	0.45820062	0.31776445	0.71587074
0.36793644	0.59672706	0.24325449	0.51540714	0.36592836	0.52016146	0.76046095
0.37564322	0.39625156	0.0854081	0.49588069	0.34015289	0.26783031	0.68016481
0.41872705	0.48901802	0.17215239	0.46918091	0.31491645	0.28767423	0.68548794
0.347439	0.52968187	0.18075079	0.49779446	0.30217572	0.33540826	0.72342958
0.37815913	0.51120837	0.21759748	0.51714389	0.33544862	0.3694646	0.69796619
0.44093843	0.51904536	0.20942981	0.50584067	0.34603322	0.22328386	0.66612397
0.33673724	0.59577945	0.38547793	0.53264387	0.36622237	0.65008427	0.77214828
0.4310833	0.52234124	0.21367299	0.52996864	0.4069437	0.53659381	0.74193327
0.39040704	0.55803598	0.3685116	0.5132306	0.52259029	0.66681439	0.77183793
0.42077926	0.5219255	0.18138323	0.51928198	0.40258245	0.44352662	0.70400031

Tcm	Tem	Tfh	Tgd	Th1	Th17	Th2
0.64184198	0.50768436	0.36450497	0.21484563	0.3828059	0.55810582	0.50725738
0.66059322	0.55674627	0.45895753	0.39050691	0.39943955	0.45690429	0.51582376
0.63136378	0.52792907	0.46334351	0.13800666	0.35206508	0.54106207	0.48914737
0.6132953	0.57207338	0.45291589	0.25319033	0.33099436	0.52627427	0.56307747
0.61804917	0.57079441	0.4375807	0.33678793	0.39568355	0.46371497	0.53015658
0.66800187	0.62918553	0.45405363	0.33705979	0.39978804	0.49369376	0.53984417
0.62041535	0.60496191	0.47203312	0.21743361	0.36730635	0.49721224	0.5138631
0.65930984	0.65666255	0.58539123	0.40799833	0.51366041	0.26563183	0.59409485
0.61637558	0.5908947	0.51405863	0.41797516	0.479313	0.46219416	0.50861154
0.63040639	0.57970253	0.44250854	0.28869636	0.39448683	0.57204008	0.48689883
0.60417818	0.60526119	0.45860191	0.46606868	0.41942216	0.52419682	0.51999095
0.64150405	0.57262944	0.41052066	0.32718266	0.38965683	0.50341303	0.4739159
0.59725766	0.57211253	0.44011138	0.17496412	0.38922884	0.50999898	0.45948882
0.57101824	0.54748059	0.46726475	0.23061998	0.34447413	0.48357591	0.49173626
0.63630441	0.59828852	0.46385268	0.29766933	0.38398299	0.5101633	0.46291375
0.64086722	0.55250758	0.45200244	0.18504123	0.32600427	0.49421179	0.52074001
0.60583396	0.58865676	0.42396829	0.21922348	0.31748624	0.56476115	0.47669184
0.60325276	0.56471322	0.47673204	0.22543882	0.37709117	0.55979115	0.56909754
0.60362014	0.61452886	0.49924172	0.13074801	0.38939984	0.37628998	0.53507257
0.62050074	0.55089926	0.50015497	0.26208986	0.38487029	0.28824951	0.58130822
0.58746509	0.60909817	0.50920028	0.17661323	0.49243172	0.45128498	0.54912089
0.59806947	0.52951914	0.50248082	0.31722508	0.3638231	0.53067905	0.54805911
0.61811725	0.57959632	0.45427906	0.29960021	0.41728831	0.54426191	0.48034379
0.59790152	0.5876835	0.48212782	0.10022847	0.3405328	0.40805105	0.50213562
0.60103701	0.56395517	0.45284349	0.31937073	0.37516492	0.53162914	0.51375452
0.56661262	0.5445403	0.47911728	0.20979175	0.41012985	0.52064182	0.53846986
0.61230705	0.50132249	0.4891852	0.2136507	0.37102263	0.36213455	0.54486241
0.6163546	0.58246666	0.47580312	0.35215198	0.3731835	0.55516643	0.48413473
0.60004365	0.51879108	0.47186093	0.23321819	0.35678944	0.47168987	0.56520011
0.57488588	0.49668188	0.44778435	0.25736713	0.34656584	0.54237998	0.58606363
0.60155158	0.56186127	0.41329035	0.23551409	0.37708481	0.5367951	0.5118491
0.67033426	0.59399081	0.45496268	0.19569993	0.37959059	0.42105721	0.49731329
0.62429723	0.58493645	0.42879729	0.29141648	0.39732077	0.51452908	0.52572012
0.64486176	0.5558769	0.52955691	0.30005135	0.41828494	0.22288407	0.53544215
0.60429383	0.56806175	0.47812573	0.20920328	0.37840376	0.43019722	0.57118948
0.65416231	0.57585889	0.48524713	0.33160734	0.44187269	0.51023702	0.55468793
0.55978004	0.56897664	0.51847638	0.2183873	0.46220515	0.2433562	0.57723756
0.63068069	0.53428	0.46614543	0.31505226	0.3958383	0.4863694	0.54650688
0.64023417	0.58692016	0.46427024	0.22406821	0.38685821	0.35640127	0.57804429
0.60610276	0.55546937	0.42185585	0.24773069	0.32207878	0.52633678	0.52835568
0.57947411	0.57013368	0.47435544	0.27633277	0.39698932	0.53993103	0.45012818
0.61502949	0.53687786	0.43898768	0.23909467	0.37078596	0.5351732	0.54437594
0.6337132	0.53405294	0.46526687	0.261354	0.43213078	0.26012667	0.5735851
0.64123743	0.60280189	0.45210801	0.43292607	0.40039582	0.53135096	0.52346633
0.66449274	0.61176821	0.44957228	0.20622629	0.41985035	0.49630656	0.52821098
0.63145822	0.57026568	0.49987335	0.29695532	0.40658323	0.57630778	0.53617491
0.59841743	0.5393176	0.50228024	0.14542788	0.33716245	0.5608569	0.48499695
0.63013262	0.53850402	0.46247653	0.48826564	0.38664762	0.51205794	0.56967331
0.62158873	0.52880414	0.40818975	0.31741654	0.38969993	0.54644455	0.50227101
0.56577975	0.52355475	0.45201891	0.21521491	0.39412799	0.51968538	0.53127522
0.61171635	0.52872584	0.43519098	0.27132247	0.31909128	0.57871873	0.51146903
0.64014616	0.53237579	0.47733076	0.19289823	0.39245834	0.51710943	0.50677197

0.62020199	0.56189274	0.42240782	0.19537576	0.33817938	0.60298037	0.50823766
0.65774607	0.58225168	0.5046538	0.34218236	0.47194284	0.51232403	0.50523999
0.58229956	0.55979521	0.45528037	0.23628376	0.39397397	0.50259527	0.48902774
0.61817673	0.57793534	0.47314322	0.32189638	0.38351434	0.52455173	0.51925569
0.62031972	0.553259	0.46502903	0.22891444	0.38453618	0.41544579	0.56773103
0.63978079	0.50856112	0.38427181	0.16517532	0.3429142	0.53382568	0.53037609
0.63240133	0.61599192	0.44822735	0.30370549	0.35269365	0.49306473	0.50234191
0.63685395	0.6162553	0.44800953	0.30472476	0.44883284	0.55648376	0.49097955
0.60552836	0.56471023	0.39187749	0.1002472	0.28873329	0.49338158	0.43924647
0.57332246	0.55012927	0.39965115	0.21768801	0.36188501	0.51172468	0.45687987
0.59693512	0.57157713	0.4555133	0.3147597	0.33897938	0.53071498	0.49338316
0.59157121	0.57396581	0.41582773	0.22929239	0.34903917	0.5400667	0.47631346
0.58749979	0.56619769	0.46720748	0.42694459	0.39382489	0.48219023	0.53827175
0.60150145	0.57396014	0.51583351	0.17443461	0.38712587	0.50234598	0.55870185
0.62037148	0.56854206	0.43088283	0.21951698	0.40414409	0.50265458	0.54199199
0.57492696	0.52674198	0.42693305	0.31230601	0.37833183	0.53581268	0.54750833
0.61367567	0.56788285	0.44989039	0.22099409	0.403067	0.5579539	0.52687342
0.60322382	0.53166733	0.4194362	0.30194913	0.36498317	0.53629794	0.50735014
0.62942852	0.53842725	0.43749949	0.13285461	0.35332971	0.4885915	0.50831859
0.604045	0.55723134	0.42172675	0.23497669	0.2915516	0.5281439	0.53834791
0.5880485	0.56979174	0.45941503	0.55753637	0.42966574	0.52630348	0.58328735
0.58619577	0.53914678	0.42073501	0.21941983	0.38936295	0.54445451	0.55009005
0.56922184	0.5228436	0.40908318	0.34580467	0.35048623	0.53826173	0.49964677
0.58447824	0.51875432	0.44546455	0.37504685	0.38466693	0.41059325	0.50330505
0.58486281	0.5449514	0.46139082	0.22743506	0.39395332	0.52028639	0.53181157
0.57811907	0.58165344	0.42889123	0.30385557	0.37428029	0.54283098	0.49515682
0.61331599	0.55887455	0.45227727	0.32034934	0.39600087	0.5374953	0.47364365
0.61533309	0.59536955	0.44120888	0.26827724	0.42422941	0.54184414	0.48937776
0.55792603	0.54175345	0.43062912	0.24721212	0.41258517	0.55683057	0.54189853
0.6270708	0.52303576	0.4801029	0.21197494	0.35962081	0.56003415	0.55102101
0.61583826	0.58339381	0.49125044	0.30321493	0.43411633	0.47494074	0.55579952
0.54198821	0.52654323	0.423388	0.20625624	0.24822945	0.52453177	0.47069846
0.62404711	0.62545155	0.42784291	0.18285299	0.35006443	0.51450526	0.43959922
0.62210195	0.58111394	0.46454793	0.2446284	0.36298495	0.50040583	0.47498634
0.55999047	0.50845521	0.39974082	0.10327021	0.37919038	0.53792265	0.46183939
0.60712925	0.60391404	0.45443301	0.3208936	0.38803857	0.51730027	0.44851822
0.59904614	0.5525357	0.42825881	0.24008745	0.34627346	0.53660454	0.57314813
0.65208948	0.59247448	0.5532755	0.20194281	0.42330792	0.2663495	0.57239565
0.60486436	0.53826963	0.44659928	0.12090761	0.30962792	0.49144949	0.52685255
0.66587802	0.54535069	0.50636404	0.19289682	0.3694352	0.51637453	0.51351361
0.61598877	0.59448841	0.49889981	0.13419176	0.3816773	0.32083184	0.50668637
0.59568146	0.56486479	0.46201076	0.27882737	0.43440664	0.58717512	0.50402484
0.58296175	0.52493932	0.39483074	0.17285633	0.31658686	0.53147246	0.49855495
0.60248182	0.56463762	0.46995411	0.23742266	0.37069426	0.49872885	0.50966154
0.636362465	0.542972271	0.433793986	0.236072216	0.36113497	0.510710906	0.45604577
0.60847531	0.6263916	0.52763017	0.39144862	0.37047246	0.52719185	0.46423695
0.62034195	0.58418803	0.54670192	0.34045471	0.46060935	0.48706959	0.56135156
0.6004238	0.60030722	0.46640335	0.37866389	0.37943683	0.57089056	0.51404729
0.56333753	0.57124094	0.49667236	0.29435164	0.39641333	0.57912693	0.57104219
0.64522715	0.53116584	0.41002394	0.23390051	0.34387572	0.53987977	0.49848227
0.53773125	0.52601193	0.45598777	0.08614274	0.32976476	0.54918175	0.46301285
0.54363301	0.55931729	0.44720022	0.35302488	0.35432302	0.50111782	0.47889155
0.6176338	0.56101104	0.4326049	0.24689983	0.36233734	0.55217251	0.50824314
0.56074641	0.51767686	0.45474863	0.31102427	0.34642432	0.5939556	0.4849909

0.58006034	0.58233872	0.44695781	0.24784197	0.29652767	0.49451243	0.45211596
0.57926154	0.56001854	0.47804866	0.20259483	0.33045026	0.47409465	0.44344384
0.59433884	0.52135075	0.48807022	0.21334249	0.37180299	0.3552336	0.5225704
0.57808223	0.61168029	0.45388618	0.16644177	0.33791074	0.52407496	0.48776682
0.59638624	0.6225354	0.50657469	0.2824928	0.42610519	0.54330867	0.49484613
0.60259651	0.5727517	0.45948835	0.38562287	0.41672985	0.46162569	0.4801044
0.61716224	0.61877713	0.47479514	0.2972476	0.39588689	0.51439574	0.45510992
0.65311276	0.5911055	0.49606973	0.20156751	0.42967922	0.49627218	0.51296198
0.63704285	0.57515095	0.45246558	0.1536529	0.36550168	0.54092293	0.51594466
0.62481309	0.54003937	0.4510644	0.14490219	0.34502079	0.52407863	0.54394744
0.66940305	0.60717581	0.51055154	0.22243807	0.41300085	0.51732092	0.48888415
0.61682798	0.55793316	0.43328987	0.16719325	0.28684883	0.56822639	0.49903111
0.63188119	0.58374697	0.4891452	0.1040254	0.30136452	0.43466477	0.4964728
0.61099293	0.63585791	0.49340473	0.19225857	0.32372402	0.50660519	0.47416426
0.639033	0.56638492	0.42954997	0.09606058	0.39923583	0.51320346	0.4981364
0.62369221	0.51324932	0.38955176	0.20828271	0.31963802	0.52158765	0.38749103
0.58946845	0.56170196	0.45793359	0.24173553	0.27664157	0.51524785	0.44828583
0.61876913	0.55433539	0.4862281	0.24577901	0.38360865	0.53355391	0.48000494
0.63292935	0.56523747	0.46050784	0.14682698	0.37023929	0.41074616	0.46606636
0.61758183	0.57660828	0.42894847	0.13000701	0.34214251	0.5483867	0.45767318
0.56377242	0.60642424	0.47742133	0.31062667	0.38661426	0.43091321	0.52178083
0.65149371	0.60650447	0.42881731	0.24351745	0.28659452	0.52672263	0.44005115
0.60827782	0.60986709	0.43362157	0.1917046	0.34795758	0.53856121	0.49275577
0.61572505	0.63603657	0.44877716	0.17050604	0.3817309	0.48527546	0.54115271
0.60104706	0.54824587	0.41517766	0.11292358	0.31457342	0.51069053	0.49214485
0.59016102	0.54117287	0.46045855	0.26951093	0.3378218	0.54206139	0.57002503
0.60002232	0.53900716	0.44128391	0.2913225	0.34518976	0.53665841	0.46303634
0.60910111	0.57895639	0.42458146	0.40645917	0.34011346	0.53540963	0.50881907
0.64289623	0.6057966	0.46075384	0.25098845	0.35803525	0.52656473	0.45903077
0.58880669	0.5382498	0.4049334	0.10806872	0.2784643	0.41697279	0.55168399
0.57011078	0.49400909	0.47818186	0.07268779	0.3392164	0.20263284	0.45494542
0.60840306	0.58792294	0.41085951	0.27404788	0.35262907	0.55391646	0.45028631
0.61921948	0.53896442	0.41863244	0.21164037	0.33733006	0.55283728	0.47056311
0.62042555	0.56361918	0.38751499	0.17160833	0.32683763	0.52669308	0.40945652
0.62111322	0.59141287	0.48089865	0.06752382	0.39582108	0.34445974	0.52249189
0.59607932	0.51093926	0.42743868	0.08895463	0.34376104	0.42811917	0.51200301
0.62010037	0.54647264	0.37696612	0.14138172	0.26083849	0.46753233	0.44325199
0.56576264	0.49713367	0.41131848	0.27109788	0.32201104	0.52725237	0.48946571
0.56329702	0.54775059	0.39918401	0.19969572	0.3354657	0.52929196	0.41689637
0.56556334	0.54054533	0.44364048	0.36654605	0.26274945	0.55510266	0.44892532
0.66146019	0.62747867	0.51188038	0.17886238	0.38210841	0.51065608	0.49874171
0.64125894	0.5653182	0.47908861	0.21596649	0.37592365	0.51934451	0.43416072
0.59387097	0.56787896	0.52253351	0.27146859	0.4377878	0.51061528	0.49502369
0.60330279	0.60173112	0.40139501	0.15071656	0.29603459	0.49387373	0.46844327

Treg	Activated_CD	Activated_dei	Anti-inflamm	CD4+_regulat	CD8+_T_cells	Cytolytic_acti
0.24935071	0.41158509	0.50108929	0.20022933	0.30407839	0.28332982	0.39661212
0.36293919	0.50262816	0.539573	0.27097659	0.27529161	0.52396841	0.6250387
0.22127211	0.45826848	0.52134577	0.16070424	0.20940023	0.44944212	0.46439946
0.62118391	0.53184608	0.55493509	0.19151977	0.2919281	0.41804719	0.55336109
0.47853678	0.52355281	0.55567415	0.27554548	0.31051241	0.51847744	0.57100807
0.09582496	0.5376776	0.57626207	0.14385024	0.28208329	0.49693272	0.60970074
0.32672612	0.48468221	0.55230052	0.20984575	0.27166444	0.37294579	0.49481689
0.44854707	0.64040916	0.5934219	0.36486154	0.33576417	0.82555256	0.78263503
0.53895722	0.58211398	0.58431923	0.26315251	0.33837671	0.74717002	0.71734667
0.30050963	0.50322886	0.56460808	0.2084383	0.27789074	0.44667568	0.4960948
0.46050375	0.52603001	0.5758869	0.26461837	0.32835901	0.48347775	0.58901358
0.31197628	0.48413052	0.53027329	0.24160722	0.27831799	0.34955678	0.43956714
0.12890185	0.46840745	0.52220346	0.21718712	0.22441179	0.33446709	0.38556121
0.08298623	0.45258927	0.51578757	0.19018548	0.1816346	0.15150464	0.37187518
0.20823737	0.42927	0.52479956	0.24180346	0.26543079	0.39017319	0.52097182
0.01918459	0.49703089	0.51889996	0.1681503	0.22718629	0.33828143	0.40051098
0.13973146	0.5113444	0.52545863	0.1823395	0.25613508	0.33538924	0.36300965
0.28987602	0.56677635	0.55069909	0.19005681	0.30017682	0.35123341	0.41389645
0.28330965	0.53416037	0.54804521	0.48539537	0.22024904	0.24828822	0.49858357
0.35103151	0.53430387	0.53880536	0.32828743	0.26835116	0.30533562	0.44084089
0.46153281	0.55392666	0.57799703	0.3123323	0.32294426	0.46889105	0.58432527
0.12694174	0.46060204	0.52606122	0.2024147	0.22840833	0.18440854	0.3121891
0.24694958	0.42448347	0.55297991	0.26626665	0.27925825	0.37135299	0.49254717
0.21779291	0.51574849	0.51461164	0.22892806	0.2086914	0.24627626	0.25395896
0.28634782	0.49554429	0.520536	0.17316727	0.24782858	0.32822164	0.40520381
0.36935855	0.53422241	0.56031857	0.27890023	0.29225144	0.32050913	0.39510513
0.13350811	0.52920004	0.5069496	0.14617752	0.23313307	0.41628673	0.40345638
0.11371098	0.44354803	0.54540301	0.21743428	0.23190316	0.3902151	0.5404157
0.28968001	0.49635688	0.53506661	0.11742867	0.26083024	0.35223939	0.31841651
0.45530945	0.49835644	0.52154435	0.17997591	0.28913736	0.13260062	0.33259285
0.50651737	0.48689149	0.5489161	0.20162732	0.27380535	0.43129258	0.41688988
0.39719214	0.4656895	0.55219922	0.17330527	0.26546359	0.39591565	0.503069
0.84463664	0.48193713	0.56584826	0.25095316	0.35642751	0.47266347	0.50832617
0.37940413	0.53548743	0.54090816	0.27011576	0.27551279	0.48305859	0.48246821
0.17060322	0.53800041	0.55720962	0.15529882	0.29154239	0.45484926	0.43125083
0.18790121	0.55986441	0.55618713	0.21170463	0.29901793	0.76242737	0.74209585
0.34706228	0.61024628	0.5320649	0.2328596	0.30304311	0.69154776	0.66496444
0.40664968	0.56050949	0.54871279	0.21417117	0.28427984	0.39545458	0.50507731
0.37989415	0.57474912	0.56481877	0.30022579	0.31561231	0.50129196	0.51936622
0.48387808	0.44874982	0.51277464	0.29320668	0.26067695	0.28756332	0.29029256
0.33760474	0.41745052	0.56429193	0.26957269	0.29140587	0.44713675	0.57907057
0.25145783	0.47468697	0.50566245	0.15641241	0.22747373	0.30990444	0.413633
0.38905768	0.55781299	0.53442936	0.20703304	0.31250854	0.48025023	0.51358136
0.40272946	0.51032659	0.56090353	0.38805944	0.32921133	0.61027303	0.62899586
0.17658157	0.48674518	0.55420083	0.30554257	0.28445407	0.37835293	0.39587771
0.97072083	0.44709806	0.55223916	0.26974226	0.45199601	0.43925658	0.47801965
0.21793992	0.50217841	0.49347018	0.23723788	0.22438314	0.44210686	0.41910022
0.21789092	0.53681641	0.5455116	0.20248747	0.25671496	0.25776119	0.39428681
0.13615426	0.48697967	0.53529291	0.17763864	0.26720767	0.42202919	0.45802282
0.36063606	0.48056627	0.54590024	0.25524503	0.256011	0.46243602	0.50190878
0.29874553	0.46764912	0.4783889	0.13565276	0.24803475	0.14295382	0.27272447
0.27845837	0.52705301	0.52044668	0.24247751	0.24443912	0.48942979	0.48653848

0.19412457	0.45524422	0.5255113	0.217914	0.22383308	0.28865313	0.37303842
0.45134023	0.53243035	0.55749437	0.27363742	0.31100752	0.76485849	0.69633991
0.41351007	0.51532865	0.54870385	0.27095544	0.27360337	0.46537012	0.51390029
0.58198167	0.46832073	0.53813988	0.29957827	0.32218288	0.48553162	0.52997248
0.31413241	0.58112497	0.51187722	0.2395906	0.31535805	0.34867655	0.3243813
0.1806978	0.44961993	0.50792529	0.15697275	0.19756362	0.3143056	0.33596289
0.29629539	0.53010886	0.55634886	0.23959688	0.26632782	0.55599208	0.56657897
0.36984858	0.52828631	0.57525674	0.23663019	0.3119134	0.5629501	0.60428203
0.27110795	0.40107929	0.45389419	0.05823825	0.22091977	0.14756456	0.34458265
0.18491204	0.43656589	0.51228331	0.11286124	0.24429916	0.33375453	0.35572758
0.74775812	0.39802232	0.52255128	0.25801824	0.26154577	0.41565799	0.55643206
0.22489832	0.47380406	0.52994797	0.209448	0.24903772	0.36510754	0.47688405
0.37871809	0.51527692	0.52469401	0.20123682	0.26361478	0.54048323	0.46741893
0.21833194	0.51358552	0.54349194	0.25328345	0.22940055	0.53520184	0.50962202
0.29747146	0.56389432	0.54910413	0.30111926	0.28147654	0.43711888	0.49122167
0.26272848	0.52906989	0.54254473	0.20454768	0.30338209	0.37998765	0.3915565
0.67993826	0.40423415	0.53821319	0.22233259	0.35036718	0.40987361	0.47837612
0.85825942	0.40331578	0.52918103	0.30781736	0.33150972	0.4079874	0.40753833
0.13786936	0.51085362	0.50621598	0.22681061	0.19935048	0.35207173	0.35446733
0.58693096	0.50354615	0.49586033	0.32444481	0.41095899	0.19258212	0.38438396
0.38518646	0.57198184	0.56093055	0.22277456	0.31640772	0.38392773	0.40410369
0.2946783	0.4895998	0.56429418	0.24556419	0.27368695	0.33958082	0.44087821
0.26130739	0.45973941	0.54175614	0.16538251	0.22009347	0.27469517	0.37799366
0.36181212	0.47486528	0.56098558	0.20734551	0.25706705	0.22946803	0.31927516
0.11287793	0.49168618	0.53472096	0.29010548	0.25170433	0.37462243	0.40569463
0.15683344	0.50072337	0.55584733	0.26637718	0.22909778	0.48339392	0.54833862
0.37352379	0.48498155	0.54157569	0.27679152	0.28615303	0.44483138	0.53369896
0.2649826	0.49706943	0.55339482	0.30238538	0.26913559	0.54140538	0.53934124
0.27429313	0.51385038	0.57108435	0.25505096	0.27079637	0.27205447	0.29094601
0.54361249	0.48723501	0.53363728	0.23747086	0.31970177	0.35232322	0.41928239
0.36191013	0.57546328	0.59918086	0.27989175	0.30989121	0.64137455	0.58666038
0.99914245	0.42869343	0.503186	0.20329425	0.30950223	0.33618564	0.39617264
0.37587592	0.44523577	0.55939877	0.26205638	0.25297272	0.48930404	0.55885648
0.89540354	0.40666695	0.54205896	0.20767344	0.3368517	0.41498734	0.50823106
0.38822463	0.43306805	0.51149881	0.14953379	0.27518573	0.35374836	0.44091184
0.21098153	0.47103531	0.5291264	0.20644909	0.23081128	0.51696847	0.53211626
0.24400941	0.44872689	0.49676009	0.12452772	0.20766472	0.3198804	0.40203128
0.69669721	0.56668245	0.53172423	0.31915766	0.39585848	0.63936259	0.58312927
0.38489244	0.50848849	0.49446119	0.09168774	0.20829343	0.4491068	0.45005561
0.29605037	0.47374546	0.52631908	0.19641266	0.28033639	0.33425752	0.38379775
0.33990787	0.47406946	0.49295003	0.31570459	0.31292737	0.37705354	0.31807566
0.34858137	0.48541633	0.55524859	0.3023867	0.35124022	0.60964429	0.48046625
0.36122409	0.45027436	0.48380035	0.1001156	0.23923452	0.33333537	0.35424868
0.27581222	0.47410475	0.57798715	0.2404966	0.26443134	0.41033468	0.49978448
0.362694174	0.440567204	0.53916869	0.227089862	0.257873828	0.408029316	0.46205883
0.44962513	0.49172934	0.54029723	0.27638224	0.29173308	0.71946368	0.67938906
0.412285	0.62784666	0.56195283	0.24386826	0.30628368	0.81503169	0.76589508
0.5262655	0.43889124	0.5473172	0.26960467	0.35359801	0.45287922	0.56141179
0.32682413	0.48641824	0.55176471	0.39070669	0.32673934	0.2660186	0.4402327
0.54763071	0.46929899	0.50161485	0.24860385	0.30170502	0.42714292	0.38218466
0.16335081	0.40205608	0.50101811	0.2100295	0.17852246	0.19325278	0.1154478
0.21416671	0.45100166	0.53611162	0.21640759	0.21168494	0.24459963	0.35971166
0.75456951	0.47167719	0.53475918	0.22568891	0.39833557	0.46859764	0.49857633
0.21838095	0.37848273	0.52683453	0.24427728	0.30441562	0.41150833	0.35549552

0.93436076	0.3680829	0.50723677	0.18280324	0.34261711	0.38472413	0.47129237
0.2842897	0.42187803	0.4977758	0.2350795	0.1951906	0.28957528	0.28370575
0.36249816	0.47476918	0.5210662	0.32245017	0.31742866	0.37017935	0.38497349
0.25689714	0.50385516	0.55518545	0.27155298	0.26729175	0.379317	0.38229445
0.40846278	0.53080781	0.55747357	0.27728064	0.30354592	0.5321839	0.48266625
0.46153281	0.49982172	0.56951543	0.27872613	0.27949471	0.37319729	0.4675594
0.30442985	0.4636004	0.54080094	0.19968164	0.22935639	0.55750104	0.54923817
0.47530259	0.50249446	0.56172086	0.22249243	0.34958548	0.73539168	0.71167271
0.31506346	0.40668253	0.53089376	0.23995913	0.27407005	0.37378411	0.40241151
0.29943157	0.53580862	0.48162996	0.12819119	0.25536398	0.47568141	0.43317702
0.52508943	0.47051987	0.53342013	0.21626625	0.30639193	0.62050049	0.5772963
0.53895722	0.39745828	0.52371414	0.13559218	0.24403312	0.29431176	0.40344075
0.30648797	0.49647153	0.49015112	0.17430588	0.22417257	0.40111321	0.29416546
0.25410398	0.45905174	0.49246244	0.25937045	0.20848179	0.39696355	0.44936245
0.43482629	0.48205915	0.51465389	0.09420455	0.25817403	0.40916104	0.38582089
0.43693341	0.36449851	0.48199589	0.13244121	0.26052155	0.24275533	0.3772158
0.25248689	0.43083662	0.53084238	0.16485298	0.21632884	0.52811807	0.53369138
0.73540942	0.43163184	0.54029106	0.20673239	0.34306243	0.57300989	0.54779027
0.37494487	0.46478449	0.50557493	0.22998552	0.24547731	0.51797445	0.44976115
0.2648356	0.44017919	0.49914239	0.25129324	0.24276486	0.24581519	0.30963939
0.40542461	0.49597831	0.55961342	0.2796644	0.25386746	0.43481351	0.45043147
0.11812123	0.45561028	0.52748694	0.15544608	0.24059263	0.34649693	0.48958816
0.30271475	0.45747466	0.54327533	0.15080535	0.22751624	0.37730504	0.43135249
0.3297643	0.53373475	0.51368719	0.22079548	0.21678363	0.39331687	0.3710693
0.25498603	0.53382349	0.51921093	0.198019	0.26882012	0.35928125	0.34115411
0.33221444	0.50110642	0.52612993	0.18998522	0.26200392	0.39956233	0.38373214
0.78715637	0.44751485	0.53482448	0.20527515	0.34094705	0.49160941	0.54806026
0.2754202	0.4433416	0.51048538	0.23646847	0.25433991	0.18579177	0.32133434
0.32966629	0.46873608	0.53140122	0.24814357	0.30987931	0.52585462	0.52728442
0.22558436	0.47990233	0.49727168	0.14482214	0.17257657	0.11164272	0.32768081
0.3701426	0.48859591	0.49003798	0.13916104	0.23316203	0.44416073	0.45008592
0.2911991	0.44862025	0.51848731	0.26131314	0.30490124	0.40203536	0.40994655
0.30986916	0.47210468	0.50894767	0.13596713	0.23614178	0.37592182	0.40317799
0.36926055	0.30838773	0.50352431	0.12191412	0.25480722	0.30650926	0.35008482
0.51107463	0.53318221	0.54132853	0.30094196	0.28997595	0.35974232	0.32800811
0.19971088	0.48850459	0.48914795	0.11497101	0.21031125	0.2458571	0.25430511
0.46241486	0.38047412	0.51577291	0.13289835	0.23205711	0.27905441	0.2478093
0.19799579	0.44630201	0.53201518	0.13669603	0.18116316	0.19585156	0.27077343
0.21196158	0.39823558	0.52382939	0.23344449	0.23782511	0.29439559	0.31217963
0.03814867	0.39573179	0.48485196	0.12575814	0.16004749	0.28986869	0.32089457
0.36779046	0.54564912	0.54245753	0.14854421	0.30225065	0.66660786	0.66900928
0.28649483	0.4098106	0.51238259	0.13093354	0.24947802	0.64414099	0.56043157
0.44398981	0.5631652	0.54647553	0.19359829	0.2941978	0.70952964	0.69116273
0.32706914	0.402563	0.51537424	0.17975233	0.20407759	0.34486221	0.43381934

M1_macropha	M2_macropha	MHC_class_I	NK_cells	Pro-inflammatory_cytokines
0.46284929	0.3083196	0.8198074	0.18417661	0.104677
0.71166714	0.36877442	0.88514473	0.34695021	0.13137406
0.52406245	0.3087791	0.86571821	0.07067669	-0.0068941
0.64353918	0.43895149	0.87094141	0.18055902	0.0279772
0.62316191	0.45423466	0.86444868	0.26159688	0.16610325
0.73135683	0.45630966	0.86417203	0.23146694	0.22117046
0.64688945	0.52102711	0.87341078	0.17387568	0.12220846
0.84546663	0.53011733	0.89382957	0.3107915	0.27459217
0.70765376	0.4592498	0.89397901	0.26681006	0.30493252
0.45541832	0.4001661	0.87315597	0.1570089	0.07838548
0.61977192	0.50677903	0.86569144	0.25034483	0.18353423
0.51360726	0.37335308	0.84380333	0.23898616	0.09238339
0.52318244	0.42706069	0.86591416	0.13314452	0.07510875
0.47179215	0.2719396	0.84935844	0.06896738	0.08578217
0.56769833	0.33340447	0.87470558	0.40115325	0.03463757
0.4346121	0.33223872	0.8675774	0.26745442	-0.0273834
0.5167331	0.28262977	0.85782767	0.17369197	0.00787051
0.58953892	0.39255915	0.88102137	0.19202609	0.13308588
0.612673	0.2518647	0.87259707	0.22009335	0.05426249
0.5909219	0.35392751	0.82311903	0.23317574	0.11236092
0.74616617	0.4853542	0.88691862	0.27714087	0.25762468
0.45581806	0.29618432	0.86788837	0.14591265	0.07862819
0.48613433	0.40543409	0.87412271	0.28162689	0.13644547
0.41537728	0.24933485	0.78779097	0.07625683	0.04398501
0.49403366	0.38287176	0.82588711	0.23530769	0.07204131
0.66859953	0.34627508	0.88493032	0.14876847	0.18374613
0.39995765	0.20926821	0.844574	0.09080815	0.030756
0.52282156	0.46159343	0.86654255	0.33017199	0.14320343
0.49253411	0.32485748	0.88359346	0.05001686	0.04846748
0.48529836	0.35139257	0.85177392	-0.0101648	0.02489377
0.54781957	0.34504556	0.84962283	0.16629506	0.14555054
0.496537	0.40920718	0.81920578	0.23332756	0.13208699
0.61722854	0.51023753	0.86686404	0.19324671	0.06461005
0.5573824	0.30451475	0.840807	0.19637762	0.12336604
0.66407276	0.34232116	0.88254545	0.02772382	0.20897491
0.7908003	0.33918402	0.88723319	0.42797428	0.18386082
0.75352561	0.40803671	0.88382285	0.19159615	0.1830197
0.61219732	0.39948494	0.87596552	0.25090894	0.15450034
0.6352995	0.45678445	0.8379999	0.24863724	0.25674155
0.54122272	0.36784804	0.84637703	0.15913364	-0.0196913
0.6219154	0.5335679	0.88062293	0.28288867	0.13162857
0.4034431	0.33403998	0.84553649	0.15692379	0.05697811
0.51046574	0.39389446	0.85270524	0.11232792	0.09799188
0.61099306	0.46050653	0.85725255	0.31560735	0.22122576
0.56693212	0.38763911	0.84399659	0.15933895	0.18872538
0.4065546	0.39515	0.84507675	0.32451259	0.13786406
0.45936589	0.31555214	0.87628812	0.15999049	0.04293685
0.57559657	0.34073872	0.86897749	0.22450462	0.12580989
0.5535626	0.3381677	0.84808755	0.23479682	0.09738313
0.54561271	0.35057511	0.85777781	0.0287874	0.04945659
0.35917483	0.32272137	0.82403547	0.04017915	0.02323699
0.49953346	0.25962409	0.86498793	0.25720157	0.09210086

0.45049733	0.3350123	0.83593715	0.08437207	0.04610693
0.69111314	0.34826748	0.88085441	0.25354761	0.17172652
0.64701469	0.45884281	0.87282825	0.00766171	0.07822846
0.57765049	0.46604618	0.86218451	0.13535397	0.12516514
0.55016419	0.36210714	0.84139342	0.08351181	0.04817351
0.37967059	0.36792011	0.83530425	0.20157542	-0.0161851
0.66783338	0.49317826	0.89228337	0.21585134	0.10603051
0.73401037	0.46199411	0.8802303	0.21702525	0.12930096
0.33241289	0.16901159	0.81462595	-0.0742672	0.01848179
0.51888103	0.26360319	0.83728566	0.06585095	0.07673126
0.44443612	0.37681261	0.84474473	0.35378999	0.07636911
0.48749269	0.43687994	0.84704359	0.13996313	0.06311702
0.52659173	0.33533349	0.87961322	0.11081788	0.14093466
0.62237837	0.39624768	0.89104904	0.13350285	0.22205978
0.5837518	0.40953716	0.87922594	0.2056218	0.13102573
0.5182571	0.35783949	0.88096783	0.20038573	0.06691466
0.52076856	0.38711368	0.85385667	0.28946726	0.18390857
0.48654049	0.40740216	0.83150265	0.21109596	0.08314599
0.46405626	0.35413024	0.80458092	0.05213851	0.01223767
0.28456451	0.33949841	0.86617466	0.278675	0.0890657
0.55993904	0.39831734	0.85845888	0.23940453	0.15304747
0.42547246	0.364083	0.85688102	0.17470265	0.13919654
0.34492747	0.34901809	0.8621279	0.11616073	0.09649592
0.43015329	0.35563379	0.83490917	-0.0799143	0.07114759
0.42785089	0.368435	0.84173779	0.08055133	0.08446343
0.5266665	0.4161232	0.8586233	0.23284694	0.10175278
0.58364813	0.4029654	0.89055275	0.22914809	0.14567765
0.56034346	0.43964451	0.84858763	0.25012867	0.22681095
0.42888577	0.36204086	0.85220944	-0.0283777	0.14226828
0.47515289	0.37479282	0.83014907	0.19910596	0.10744085
0.67136109	0.40949096	0.87977659	0.2373467	0.20603667
0.38052688	0.38574275	0.84027424	0.12123052	-0.029747
0.50398063	0.48009588	0.84451204	0.22709886	0.03162101
0.52444096	0.35860445	0.86931856	0.31904329	0.06279397
0.51576825	0.31104223	0.86335947	0.07379679	0.0341805
0.50459313	0.36919252	0.85267621	0.19572072	0.10978391
0.45592049	0.31414678	0.84041417	0.11936476	0.02819175
0.71807193	0.39737663	0.89222506	0.10155199	0.27413148
0.55708191	0.22437357	0.88904811	0.10118299	0.10530671
0.4455696	0.42758817	0.84177255	0.02168175	0.02385018
0.38724365	0.4394686	0.80012926	0.21013872	0.11170747
0.58783864	0.41907978	0.88288638	0.07779752	0.17165616
0.39772333	0.29461493	0.8301975	-0.0075082	-0.0022023
0.52010582	0.42418143	0.8500798	0.23516772	0.04033973
0.529836686	0.396918085	0.839621619	0.242331366	0.052176238
0.6948595	0.52822287	0.87983594	0.15134123	0.08940692
0.72470739	0.39934652	0.89402057	0.269146	0.22658474
0.45421771	0.40024571	0.84262029	0.23747718	0.11005757
0.40705787	0.3901657	0.84058503	0.12378021	0.15284609
0.52089711	0.28069031	0.84821116	0.16004141	0.01585084
0.21083627	0.27776492	0.75790145	0.12867215	0.0303754
0.50011625	0.38250917	0.86199551	0.06630225	0.03586004
0.59895452	0.35937051	0.86929793	0.23269756	0.10870248
0.43986524	0.43328531	0.85325349	0.21855738	0.15798462

0.52525127	0.38799917	0.85687804	0.11981169	-0.0024306
0.37032938	0.30796636	0.83132264	-0.0604138	-0.0981267
0.49988643	0.43422735	0.84682489	0.19773322	0.0971043
0.50382356	0.40980104	0.86732604	-0.0197312	0.01295605
0.63898511	0.4807823	0.89479464	0.23747518	0.10220518
0.66641118	0.48159716	0.85920641	0.2211743	0.1239596
0.5748777	0.38297694	0.86711453	0.19133835	0.04717157
0.66240062	0.41115219	0.89218614	0.22121379	0.1990615
0.54739837	0.32591698	0.85863074	0.11701406	0.12987353
0.48788585	0.30975811	0.86607635	0.12845089	-0.0239147
0.62320039	0.3626195	0.88070341	0.19464051	0.12893766
0.40508728	0.31433037	0.84584562	0.15458842	-0.0118533
0.35960193	0.31675738	0.83568379	-0.0133758	0.03303535
0.46755154	0.40880348	0.86176805	0.00562894	-0.008691
0.59350308	0.3822943	0.88109372	0.05961775	0.09235988
0.36452041	0.31042819	0.81600172	0.13206485	0.00062355
0.50064187	0.35824694	0.85888996	0.25930957	-0.0114252
0.5953342	0.33989324	0.87787851	0.25807826	0.09342165
0.67904682	0.30648718	0.89828426	0.1521684	0.11246057
0.32720569	0.32528166	0.81808692	-0.0733124	-0.0181279
0.57885918	0.44689554	0.86619887	0.21709702	0.0523756
0.45144626	0.40871635	0.86999145	0.17809529	0.04452261
0.61330976	0.47242501	0.88321286	0.12243355	0.03211131
0.5962997	0.3669788	0.85183708	0.09519953	0.07048139
0.54130556	0.36143443	0.86171973	-0.0449251	0.01703994
0.47686996	0.34508927	0.86717396	0.10785438	0.06250283
0.52743346	0.46319261	0.85580815	0.34368562	0.04516925
0.42869022	0.32623064	0.85180582	0.12534771	-0.0406409
0.49248907	0.36720398	0.85181231	0.28208533	0.07887186
0.28201832	0.18767661	0.79890263	0.02108413	0.01978971
0.48112042	0.377246	0.84155412	0.07555605	-0.0469075
0.37386586	0.38169928	0.85665307	0.15077507	0.04477677
0.53284786	0.21582718	0.88347317	0.19643497	-0.0222341
0.39498577	0.30559561	0.82356172	0.12985699	-0.032494
0.48864315	0.37633617	0.82463842	0.03612438	0.0819516
0.41215627	0.30948508	0.85434525	0.15824682	-0.0392103
0.32517922	0.38029163	0.85511967	0.06591412	0.00199883
0.47555348	0.30755304	0.83222772	-0.008306	-0.013614
0.41221941	0.33952209	0.8431256	0.06189902	0.05676522
0.48956343	0.34054759	0.85030657	0.16560406	-0.0328354
0.67655456	0.39181741	0.89230862	0.22093459	0.1273226
0.52192208	0.33503734	0.87544997	0.0972828	0.10128896
0.68109785	0.39871408	0.88604755	0.30629357	0.15170793
0.45810642	0.33669767	0.83606504	0.03446471	0.01951724
