# Supplementary material for: ARID1A deficiency promotes progression and potentiates therapeutic antitumour immunity in hepatitis B virus-related hepatocellular carcinoma
Source: BMC Gastroenterol. 2024 Jan 2;24:11. doi: 10.1186/s12876-023-03059-w (PMC10759659; doi:10.1186/s12876-023-03059-w)
Supplement: Supplementary file 3 — Additional file 3. Supplementary File S3. Oligonucleotides used in this study. [file 12876_2023_3059_MOESM3_ESM.pdf]

Primers for qPCR

	Sense primer(5'-3')
GAPDH	GTCTCCTCTGACTTCAACAGCG
galectin-9	TCTGGGACTATTCAAGGAGGTC
EZH2	GACGGCTTCCCAATAACA

sgRNA for ARID1A knockout

	sg1
	sg2

siRNA for ARID1A knockdown

	Sense primer(5'-3')
	GCAGGAGCUAUCUCAAGAUTT

Antisense primer(5'-3')
ACCACCCTGTTGCTGTAGCCAA
CCATCTTCAAACCGAGGGTTG
TGAGGCTTCAGCACCACT

CAGCAGAACTCTCACGACCACGG
CCTGTTGACCATACCCGCTGGGG

Antisense primer(5'-3')
AUCUUGAGAUAGCUCCUGCTT
