# Supplementary material for: ARID1A deficiency promotes progression and potentiates therapeutic antitumour immunity in hepatitis B virus-related hepatocellular carcinoma
Source: BMC Gastroenterol. 2024 Jan 2;24:11. doi: 10.1186/s12876-023-03059-w (PMC10759659; doi:10.1186/s12876-023-03059-w)

Unedited blot and gel images

The antibodies used in western blots as follow:

ARID1A (#12354; Cell Signaling Technology; 1:1,000 dilution for western blot), 270kDa

α-tubulin (#ab7291; Abcam; 1:5,000 dilution for western blot), 55kDa

The Protein Ladder used in western blots as follow:

PageRuler Plus Prestained Protein Ladder(#26619; Thermo Scientific™)

Fig. 2e

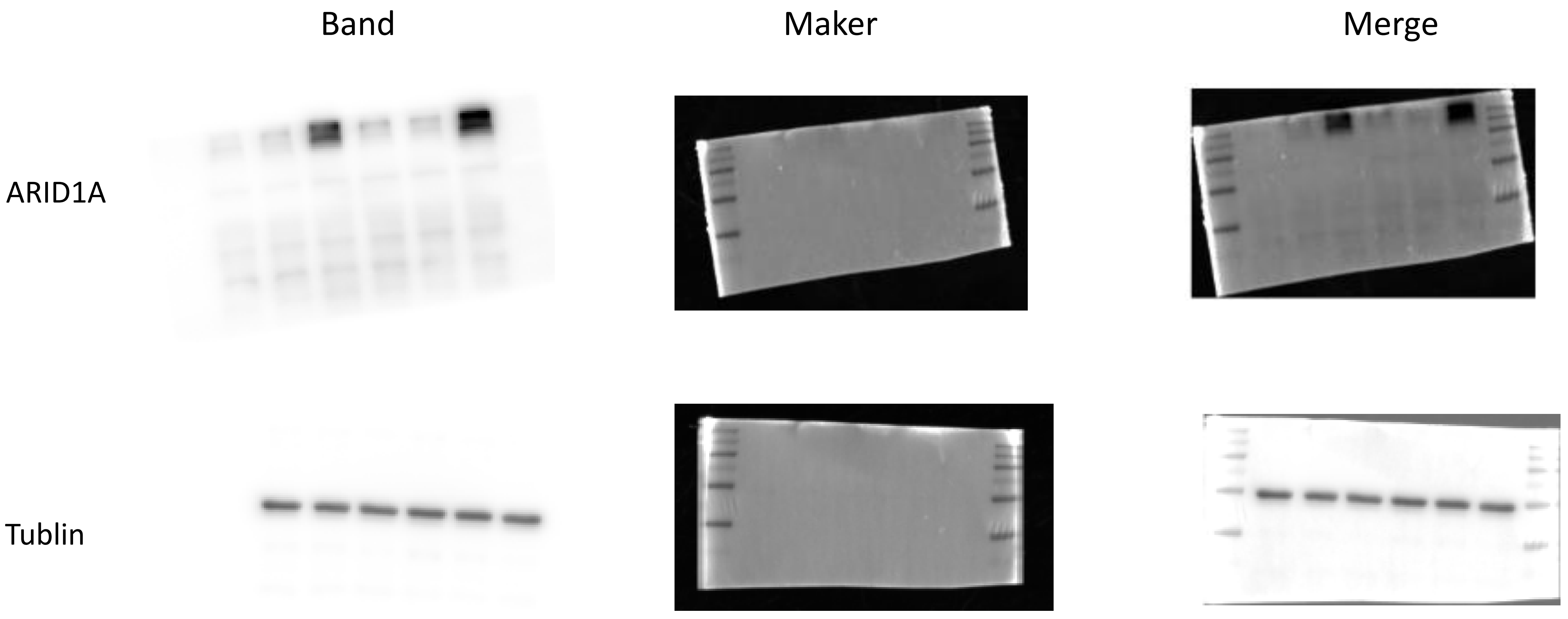

Fig. 5b

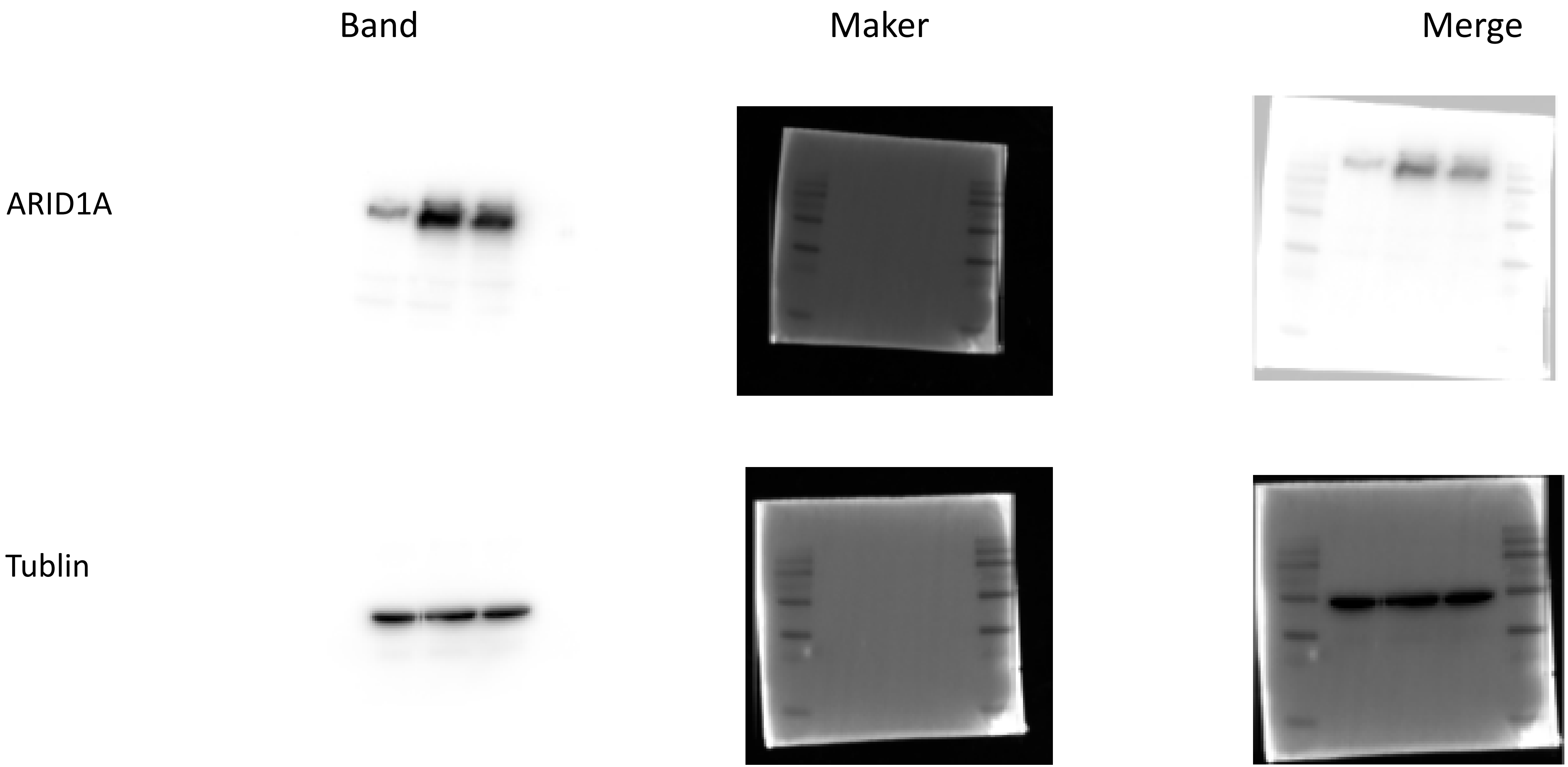

Supplement: Supplementary file 4 — Additional file 4. [file 12876_2023_3059_MOESM4_ESM.pdf]
